# Toward Self-Aware Robots

**DOI:** 10.3389/frobt.2018.00088

**Published:** 2018-08-13

**Authors:** Raja Chatila, Erwan Renaudo, Mihai Andries, Ricardo-Omar Chavez-Garcia, Pierre Luce-Vayrac, Raphael Gottstein, Rachid Alami, Aurélie Clodic, Sandra Devin, Benoît Girard, Mehdi Khamassi

**Affiliations:** ^1^Institute of Intelligent Systems and Robotics, Sorbonne Université, CNRS, Paris, France; ^2^Intelligent and Interactive Systems, Department of Computer Science, University of Innsbruck, Innsbruck, Austria; ^3^Institute for Systems and Robotics, Instituto Superior Técnico, Lisbon, Portugal; ^4^Istituto Dalle Molle di Studi sull'Intelligenza Artificiale (IDSIA), Università della Svizzera Italiana - Scuola universitaria professionale della Svizzera italiana (USI-SUPSI), Lugano, Switzerland; ^5^LAAS-CNRS, Université de Toulouse, CNRS, Toulouse, France

**Keywords:** self-awareness, affordance, human-robot interaction, cognitive architecture, learning, decision-making, planning, Markovian processes

## Abstract

Despite major progress in Robotics and AI, robots are still basically “zombies” repeatedly achieving actions and tasks without understanding what they are doing. Deep-Learning AI programs classify tremendous amounts of data without grasping the meaning of their inputs or outputs. We still lack a genuine theory of the underlying principles and methods that would enable robots to understand their environment, to be cognizant of what they do, to take appropriate and timely initiatives, to learn from their own experience and to show that they know that they have learned and how. The rationale of this paper is that the understanding of its environment by an agent (the agent itself and its effects on the environment included) requires its self-awareness, which actually is itself emerging as a result of this understanding and the distinction that the agent is capable to make between its own mind-body and its environment. The paper develops along five issues: agent perception and interaction with the environment; learning actions; agent interaction with other agents–specifically humans; decision-making; and the cognitive architecture integrating these capacities.

## 1. Introduction

We are interested here in robotic agents, i.e., physical machines with perceptual, computational and action capabilities. We believe we still lack a genuine theory of the underlying principles and methods that would explain how we can design robots that can understand their environment and not just build representations lacking meaning, to be cognizant about what they do and about the purpose of their actions, to take timely initiatives beyond goals set by human programmers or users, and to learn from their own experience, knowing what they have learned and how they did so.

### 1.1. Context and related work

These questions are not new. Researchers in cognitive science, neurosciences, artificial intelligence and robotics have addressed the issues of the organization and operation of a system (natural or artificial) capable of performing perception, action, deliberation, learning and interaction, up to different levels of development (Morin, 2006).

The term “cognitive architectures” is commonly used in the Cognitive Sciences, Neuroscience and Artificial Intelligence (AI) communities to refer to propositions of systems organization models designed to model the human mind. Among the most renown long-term projects that propose cognitive architectures with the purpose of generality, two are particularly relevant to mention in the present context: (1) The SOAR architecture, standing for *State, Operator And Result*, proposed by Lehman et al. (2006); (2) the ACT-R architecture, standing for *Adaptive Control of Thought-Rational* proposed by Anderson et al. (2004). SOAR aims at modeling human cognition and is based on Alan Newell's seminal work on theories of cognition (Newell, 1990). Operational knowledge in SOAR is represented by production rules. To achieve a goal, the rules conditions are matched to a “working memory," of which the contents is encoded as sets of attribute-values. Learning in SOAR is mainly based on a mechanism called “chunking" (other mechanisms such as reinforcement learning are being added). This process is similar to identifying macro-operators, i.e., new rules that abstract the succession of rules selected to achieve a goal.

The general concept in ACT-R is a classical rule-based system. Knowledge about facts and events and their relationships is organized in a declarative memory along with a set of production rules and procedures. The memory component contains data structures called “chunks” whose meaning is nevertheless quite different from the chunks used in SOAR. The rules associated to selecting particular chunks depend first on the existence of matching elements in memory, and second also depend on the estimated probability of success and cost of their execution. Applying these rules can result in two different operations: either trigger robot action in the world, or change the corresponding elements in declarative memory. Each chunk in memory is also associated to a “base level” which increases proportionally to the number of times they have been selected in the past. This results in using chunks that have already been selected, i.e., that were used in more successful activations of the rules. The costs and success rates of the rules is modified according to the outcome of their execution. This leads to an improvement of the global behavior through time. Furthermore, there is a “compilation” process that produces new rules from analyzing the chunks involved in goal achievement.

These two major cognitive architectures present numerous common points. First, they both employ symbolic representations at high levels of abstractions. Second, they both use production rules to represent operational knowledge. Learning mechanisms in both architectures is mainly based on a memory of the success associated to prior action execution. Neither of these architectures really tackle the issue of operating in real time, nor the issue of how to build novel internal representations from sensory data. In practice, the authors of both architectures say that these are important issues, but no clear approach is put forward to overcome these issues. Another important issue is how to link symbolic and sub-symbolic representations, which goes beyond these proposals. Nevertheless, for applications to robots operating in real-time in the world, perceiving and manipulating unprepared sensory data, this question is central.

Most previous research aiming at developing robot cognitive architectures did not address the issue of self-awareness, an expression of consciousness which is a notion that requires to be clarified, whose foundations are not proven, and which is even considered as an illusion by some neuroscientists (Hood, 2012), while others propose to ground it in the solid theoretical framework of Integrated Information Theory (Koch et al., 2016).

We want to investigate if and how a machine can develop self-awareness. By doing so, we aim at understanding the concept itself and to propose computational models that can account for it (Chella and Manzotti, 2007; Lewis et al., 2011). The paper describes how the notion of self-awareness could be related to the development and *integration* of perceptual abilities for self-localization and environment interpretation, decision-making and deliberation, learning and self-assessment, and interaction with other agents. Such an integration appears to be key to enable the robot to develop some sense of *agency*, or the awareness of being in control of its own actions and responsible for their outcome (Haggard and Tsakiris, 2009). Moreover, such an integration of the results and characteristics of various subconscious deliberative processes (such as perception, action and learning) in a common *global workspace* (Dehaene and Naccache, 2001) appears fundamental in humans to enable meta-cognitive processes such as the ability to report to oneself and to other agents about her internal state, her decisions and the way these decisions were made (Shadlen and Kiani, 2011), but also importantly to develop predictive models of agency (Seth et al., 2012).

The processes implementing these capacities must operate simultaneously for online performance in robots interacting in real-time with their environment as well as with other agents. Furthermore, central to this project is the design of an architecture that constitutes a robotic model of an efficiency-based performance testbed for the integration of these processes, and which could in a second stage be used to qualitatively (and even maybe quantitatively Oizumi et al., 2014) assess the emergence of minimal degrees of awareness as a result of their interaction for the resolution of a set of tasks. Our goal is to explore this assertion and to demonstrate it with experimental proofs of concepts.

The rationale of this paper is that the understanding of its environment (including other agents) by an agent requires its self-awareness, which actually is itself emerging as a result of this understanding and the distinction that the agent is capable to make between its own mind-body and its environment. This constitutes a dynamical system in which some authors have proposed that the awareness of self through stability and distinctiveness can be built (Marks-Tarlow, 1999; Shoda et al., 2002). We claim moreover that on the road toward a better understanding of the integration mechanisms underlying awareness, the successes and failures of robotics investigations can be useful in identifying what is not awareness, for instance when exemplifying some robotic *zombies* which can solve without awareness tasks that are thought to involve awareness (Oizumi et al., 2014).

### 1.2. What is self-awareness?

We will not attempt a strong definition of self-awareness, but we try in this paper to ground the concept. Our hypothesis is that self-awareness must first rely on perception of self as different from the environment and from other agents. This necessitates that the robot interacts with the environment and build sensory-motor representations that express the affordance of environment elements to it, and that it interacts with other agents to distinguish itself from them. Affordance building is presented in section 2 and distinction from and reasoning on other agents is discussed in section 4. Building on environment representations that integrate perception and action, two main capacities are introduced that we believe are necessary enabler of self-awareness:

- Self-evaluation. This is the capacity of “knowing that I know" and deliberately using this knowledge in action selection. I n other words, the robot builds a knowledge on what abilities it has learned and when it can use them. It is able to transform learnt behaviors into explicit skills and to characterize the situations in which these skills are applicable, reverting to a planned goal-directed behavior when they are not. This is presented in section 3.
- Meta-reasoning. The other main capacity is deliberation on one's own reasoning. In section 5, we propose a system initially driven by basic motivations, able to reason on the means for satisfying them to determine its own goals. Eventually, new motivations should be learned but this is not developed in the paper.

In section 6 the cognitive architecture for integrating all robot capacities is presented, but a validation of this global architecture still remains to be done. Finally we conclude in section 7.

## 2. Perception and learning affordances

Traditionally, in robotics perception (excluding visual servoing and similar closed-loop control) is considered only as an isolated observation process. We believe that this approach undermines the capacity of current agents (i.e., robots) for scene understanding. Simultaneously perceiving and acting requires to interpret the scene with respect to the agent's own perceptual capacity and its potential activities. What an agent can do (or *afford*) with an object partly circumscribes the meaning that this object can have for her: a mug on a table is something that can be filled with liquid and then brought to the mouth in order to drink for a human; the same object is a place on which to land (and possibly eat) for a fly, behind which to hide for a mouse, or something that can be pushed to the ground producing a fancy noise for a child. This interpretation fits with Gibson's notion of affordance (Gibson, 1977; Sahin et al., 2007).

Reasoning jointly on perception and action requires self-localization with respect to the environment. Hence developing sensorimotor representations and not just exteroceptive representations puts the robot in the center of the perceptual process, and provides a link between self-awareness and situation-awareness. Robot localization with respect to its environment provides a differentiation between the robot's body and the external world, and includes a necessary distinction between its parts and surrounding objects. In addition, robot's *actual* components link robot's body-environment's state before and after actions are applied.

In this section we propose sensory-motor representations and scene interpretation processes that integrate four inputs: perceptual (perceiving the external scene), proprioceptive (input from the agent's own configuration), contextual (previous knowledge) and the agent's action capabilities.

We propose a methodology to build models of objects based on perceptual clues and effects of robot's actions on them. Our methodology employs a Bayesian Network for representing the robot's actions, the objects in the environment, as well as changes in the observable environment triggered by the robot's actions. We then perform structure learning on continuous and discrete variables representing these informations in order to identify the most probable Bayesian network that best fits the observed data. Analyzing the structure of the obtained Bayesian network permits the robot to discover correlations between itself and the environment using statistical data.

**Figure 1:**
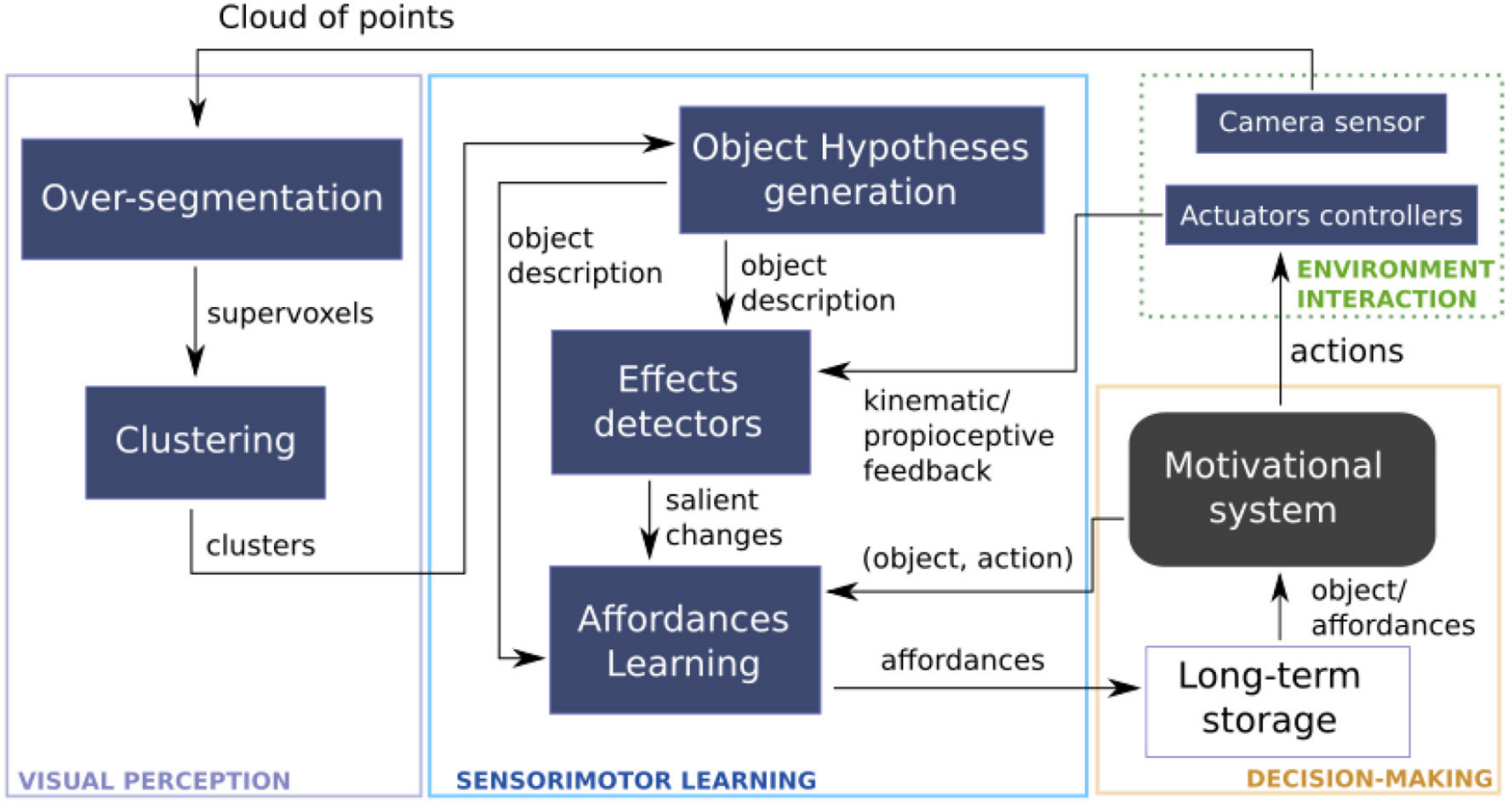
Architecture of the proposed sensorimotor approach for scene affordance learning.

The proposed affordance learning architecture is depicted in Figure 1. Measurements from the *Environment Interaction* are the main inputs of our approach, it includes visual perception from camera and proprioception values from joints. A set of clusters are extracted from clouds of points through *Visual perception*. Clusters are then tracked to generate hypotheses about the objects the robot interacts with. Proprioceptive feedback is retrieved under the form of measurements of joint and force. Then the input from perception and action tasks is analyzed by *Effect detectors* to extract salient changes from the interaction process. At the intersection between the two input processes is *Sensory-motor learning* which represents the fusion between the perception and action components. *Affordances learning* process relates *objects, actions* and induced changes considered as *effects* to build the final sensory-motor representation. A *Motivational system* orchestrates the process of selecting objects and actions that will be applied on them. The final representation is saved in a *long-term storage* which also provides feedback to the motivational system.

While interacting with the environment, the robot infers dependencies between the affordance elements (*objects, actions*, and *effects*) thus combining perceptual and proprioceptual data. The robot's motivational system relies on the learned sensory-motor representations and the Beyesian framework to make predictions about a set of affordance elements. This inferred information can be used for learning decisions, for future planning tasks, or to add sensor and motor capabilities to the innate repertoire.

### 2.1. Exteroceptive perception

To our knowledge, most existing segmentation algorithms mainly focus on raw to low level information from the 2D image or 3D point cloud. However, some recent methods for semantic segmentation have been proposed which can disambiguate object borders by taking advantage of high-level object knowledge (Silberman et al., 2012; van Hoof et al., 2014). However, the computational cost of inference on these methods rises considerably with the increasing number of objects. Moreover, the relations between nodes come from a priori information from the objects class, which limits their use in self-discovered scenarios.

#### 2.1.1. Over-segmentation

Over-segmenting a color cloud of points into small regions based on local low-level features of geometry and color enables to form supervoxels. We implemented a 3D version of the Voxel Cloud Connectivity Segmentation (VCCS) (Papon et al., 2013), which generates evenly distributed supervoxels. VCCS employs a flow-constrained local iterative clustering process which uses geometric features and color, and a seeding methodology based on 3D space. The seeding of supervoxel clusters is done by partitioning 3D space to ensure that supervoxels are evenly distributed according to the geometry of the scene. Strict spatial connectivity of occupied voxels can be enforced by the iterative clustering algorithm. This algorithm guarantees that supervoxels cannot flow across boundaries which are disjoint in 3D space even if they are connected in the projected plane.

**Figure 2:**
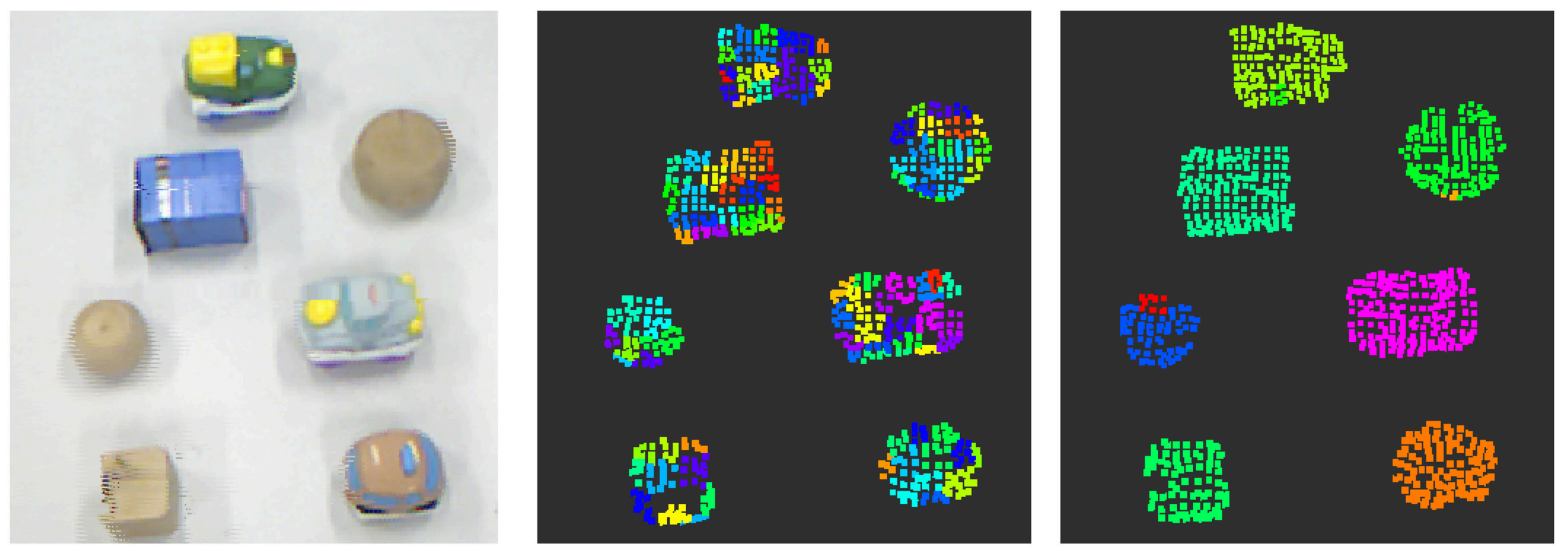
Results from the perception process. Appearance and spatial information from the RGB-D point cloud of the real scene **(Left)**; supervoxels from over-segmentation of the point cloud **(Middle)**; and results from intrinsic clustering **(Right)**.

Supervoxels are represented by a 39-dimension feature vector composed of 33 elements from an extension of the Fast Point Feature Histogram (FPFH) (Papon et al., 2013), color information (Lab color space) and spatial coordinates (*x, y, z*). This permits to exploit a pose-invariant multi-dimensional representation based on the combination of neighboring points. Figure 2 (middle) depicts an over-segmented cloud where each supervoxel (representing a segment) cannot cross over object boundaries that are not spatially adjacent in 3D space.

Supervoxels in Figure 2 (middle) only represent individual patches. A clustering process is needed to group the supervoxels that possibly correspond to the same object. The non-parametric technique described in Comaniciu et al. (2002) was implemented to find the shape of object hypotheses based on the set of clustered supervoxels.

Figure 2 (right) shows the result of the clustering method as a set of labels *L*_*hyp*_(*t*) for a cluster of supervoxels that may represent objects in the current scenario.

The set of generated segments (section 2.1.1) are built only using the sensory data. This means that segmentation issues can appear in the form of incomplete, divided and false segments of real objects in the scenario. We overcome this issue by performing a tracking-by-detection approach which reduces the number of false positive segmentations (Chavez-Garcia et al., 2016b). In this approach, each object is represented by its centroid, which additionally offers a point of interaction in further interaction tasks.

### 2.2. Sensory-motor learning

Manipulating objects enables the robot to not only perceive information, but also and most importantly to learn sensory-motor correlations between the robot's basic actions *A*, the sensory inputs contained in the objects' descriptions *O*, and the salient changes represented by the effects *E*. The objective here is to learn from regularities in the occurrences of elements in *O* and *E* when an action *a*_*i*_ ∈ *A* is triggered. While the robot is starting the learning from built-in actions, this process permits to progressively develop a representation of the environment captured by perception through object movement detection and proprioceptive feedback.

#### 2.2.1. Objects

We make the assumption that the robot has prior perceptual capabilities that enable it to discretize the environment. These capabilities are related to the segmentation approach. The robot has prior geometrical notions of position, continuity of segments and normal extraction for surfaces, can recognize different color values, and using these perceptual capabilities can extract higher level features (e.g., as combinations) for describing confirmed objects. The cloud of points representing a object can provide relevant features, such as color, size and shape. Our architecture permits to incrementally learn the set of perceptual features which are relevant in the robot's surrounding environment.

#### 2.2.2. Actions

We assume that the robot is built with a set of basic motor capabilities, or actions, described relative to the actor and its morphology. These basic actions *A* = {*a*_1_, …, *a*_*n*_} are defined with respect to their control variables in joint space:

(1) $$\begin{array}{r} a:{\left\{Q,\overset{{}^{\circ}}{Q},\ddot{Q}\right\}}_{\tau } \end{array}$$

where *Q* are the joint parameters of the robot used in action *a*, and τ the duration of this action. This implies that, by definition, two actors with completely different motor capabilities and morphologies cannot execute the same actions (but their effect might be identical).

The extraction of points of interest in the image representing a particular object is done by raising perceptual hypotheses about possible identifications of this objects. These points are used to reduce the set of possible actions that permit to approach the object through perceptual servoing. In that sense, the focus of our work is really on sensorimotor representation through object manipulation.

#### 2.2.3. Effects

An effect is a correlation between an action and a change in the state of the environment, which includes the agent itself. Effect learning can be crucial to build internal world models used for learning and decision-making, consisting in actions' effect in terms of possible rewards and possible transitions to different states of the environment (see section 3 for examples of how the robot can use such world models).

When a robot interacts with an object it can perceive (via its exteroceptive capabilities) changes related to the position or appearance of the object, proprioceptive values from actuators and feedback from end-effectors. Effect detection (or lack thereof) represents the common ground for perception and action frames. Robot's capabilities to detect effects are divided into two groups: perceptual-based (e.g., changes in perceptual representations of objects); and proprioceptive-based (e.g., changes in robot's internal representations).

#### 2.2.4. Affordance learning

We follow the definition of an affordance employed in Andries et al. (2018), where we consider *O* the set of objects, *A* the set of actions, and *E* the set of observable effects. When an actor *g*_*m*_ applies an action *a*_*l*_ on object *o*_*k*_, generating the effect *e*_*j*_, the corresponding affordance α is defined as:

(2) $$\begin{array}{r} \begin{array}{cc} \alpha =\left(\left(o_{k},a_{l}\right),e_{j}\right), & \text{for }o_{k}\in O,a_{l}\in A\;\text{and }e_{j}\in E, \end{array} \end{array}$$

This definition shows an affordance as an *acquired* relation between the elements in *O*, *A*, and *E* (Chavez-Garcia et al., 2016a).

**Figure 3:**
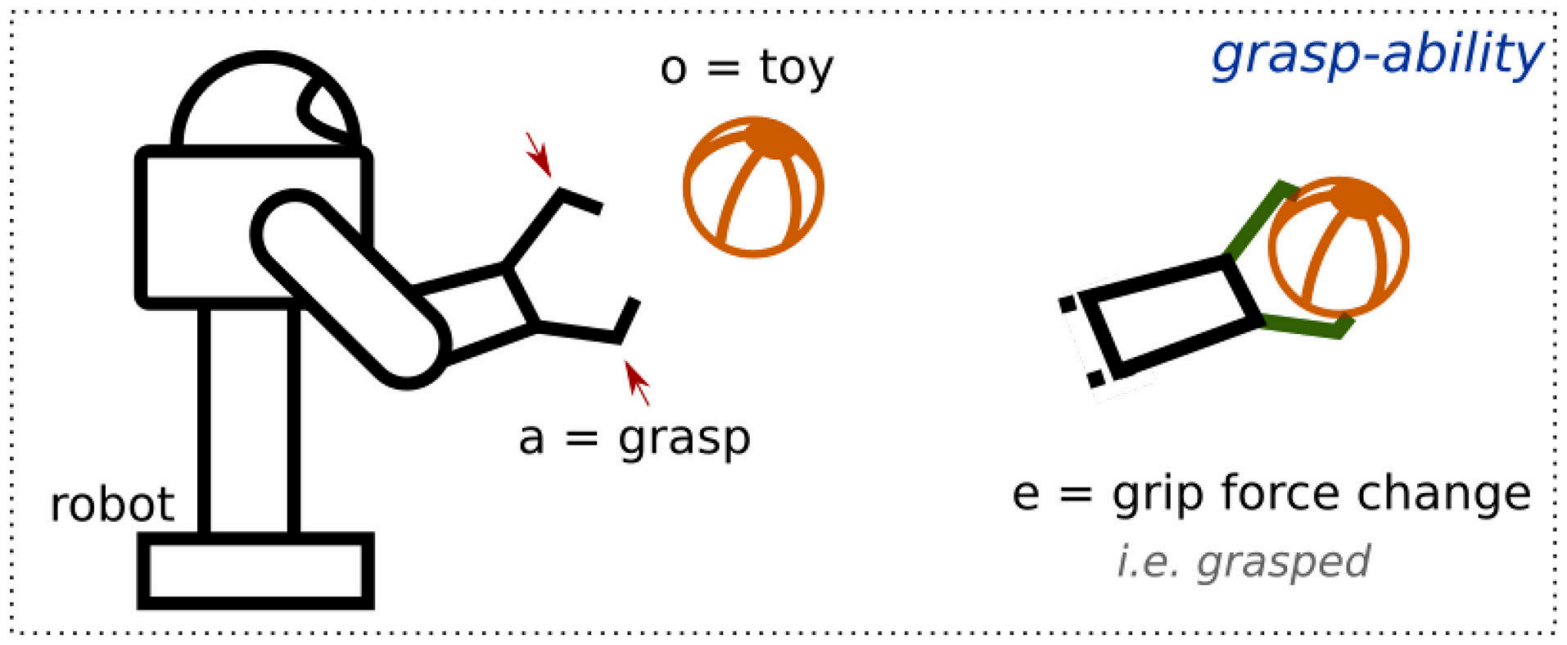
Representation of the *grasp-ability* affordance relation.

An example of an affordance relation between the object *toy* and the *robot* is shown in Figure 3. It illustrates the application of the robot's capability *grasp*, implying that there is a potential of generating an effect *grasped* that can be detected by the robot's exteroceptive and proprioceptive capabilities (e.g., grip force change). Using the semantic value of this relation, we can label it as *grasp* − *ability*.

When the robot interacts with the environment, we record the values of each element in the affordances' sets. By considering each element as a random variable in a Bayesian network B, the problem of discovering the relations between *E*, *O* and *A* can then be translated into finding dependencies between the variables in B, i.e., P(B|D), which means learning the structure of the corresponding Bayesian network B from interaction data D. In this way, affordances are described by the conditional dependencies between variables in B.

The score of a structure is defined as the posterior probability given the data D. We implemented an information-compression score that applies a penalization defined as $s\left(N\right)=\frac{log\left(N\right)}{2}$ to represent the number of bits needed to encode B (Chavez-Garcia et al., 2016b). This score penalizes structures with larger number of parameters.

We implemented a search-based structure learning algorithm based on the hill-climbing technique (Chavez-Garcia et al., 2016b). The algorithm receives as input the values of variables in *E*, *O*, and *A* recorded during robot's interactions. It attempts every possible single-edge addition, removal, or reversal, selecting as current top-candidate the network with the highest score, and iterating. For each tested structure the algorithm estimates the parameters of the corresponding local probability density functions. The process stops when the score can not be increased anymore by a single-edge change. Although this algorithm does not guarantee that it will settle on a global maximum, a simulated annealing technique was implemented to avoid getting stuck in local minima.

Such a robotic implementation of the Bayesian Network framework for perception allows the robot to display relationships between affordance elements. The directed nature of its structure approximates cause-effects relationships and includes uncertainty from the interaction process. Moreover, in addition to direct dependencies, the model can represent indirect causation. These elements are key to enable a first minimal level of self-awareness of the robot by being able to monitor the effects of its actions on the environment, differentiate itself, other agents, movable objects and fixed elements of the environment. The uncertainty about the learned effect can moreover enable the robot to display some degree of confidence about the things it learned and to explicitly require more interactive experience with the objects and actions for which it is less confident. Finally, the estimated transitions between states of the environment that can be learned within world models enable some degree of anticipation, permitting the robot to predict future states of the world depending on its actions and on the actions of the others (as we illustrate in the joint action framework presented in section 4). These capacities will be crucial for planning and model-based learning abilities developed in the next section.

## 3. Learning actions and plans

One of the main points presented in this section is that the ability to coordinate different strategies for decision-making and reinforcement learning (here considered as the main adaptation process of decision-making) can constitute a first step toward (i) more robotic autonomy and adaptation, but also toward (ii) the capacity for the robot to analyse the efficiency of its decision-making processes and use this analysis to change not only its behavior but the way it generates its behavior. Moreover, performing efficient online dynamic coordination of multiple learning and decision-making systems requires the implementation of a *meta-controller* within the robot cognitive architecture, which observes what each system does, and predicts and monitors their effect on the robot's internal state and environment. This can thus participate further to the emergence of self-awareness as integration of deliberative and reporting processes.

Here we consider that the motivational system of the robot (see section 5) provides reward to the latter when it fulfills certain tasks (e.g., recharging its batteries in a particular location, or answering a human request). We further make the assumption that for the duration we consider, this motivation will remain stable. In order to accomplish the task and satisfy its motivation, the robot needs to act in its environment. Its action selection mechanisms are then in charge of producing the relevant behavior to reach the task's goal. These action selection mechanisms have been traditionally modeled by the robotic community by action planners (see Khamassi et al., 2016; Ingrand and Ghallab, 2017 for recent reviews). Planners produce a sequence of actions to bring the robot from its current state to the goal state. Initially based on first-order logic (Fikes and Nilsson, 1971), these planners have been extended with probabilistic methods to take into account uncertainty by modeling the problem as a Markov Decision Process (sometimes Partially Observable if the uncertainty is on states). This also allows to use reinforcement learning (RL) algorithms (Sutton and Barto, 1998) to find relevant policies.

In RL, two main categories of methods can be used: model-based methods learn and use the transition and reward models of the problem (respectively the structure of the state-action-state space and the reward signals in the state-action space); model-free methods locally learn the reward-predictive value associated with each state-action pair without explicitly taking into account the effects of the action predicted by a world model of the task. The former are comparable to planning, as they find the optimal policy (i.e., the best action plan) through a costly computation using a model of the task, and hence completely update the policy between two interactions with the environment. The latter are reactive methods allowing fast action selection but are slow to learn, requiring multiple interactions with the environment to locally update each state-action value. Each type of action selection process has its advantages and has been used in a variety of applications (Kober et al., 2013). However, research in robotics have only recently started to consider the possibility of combining these two different learning methods as parallel alternative strategies to solve the same task (Caluwaerts et al., 2012; Renaudo et al., 2014).

These multiple action selection systems architectures for robotics are inspired by biological evidence of a comparable systems-combination process in mammals. Neurobiological studies have highlighted the existence of a *goal-directed behavior* when mammals are moderately trained on an instrumental task (Yin and Knowlton, 2006; Dayan, 2009). This behavior is characterized by a decision-making process oriented toward an explicit goal representation. It is moreover hypothesized to rely on the progressive learning of an internal model of the task structure, the use of this model for prospective inference and planning being experimentally observable through transient increases in subjects' deliberation time (Viejo et al., 2015). This enables a high flexibility in response to sudden changes in the task (e.g., the source of reward is moved), because behaviors that the internal model do not estimate as leading to the goal anymore can be inhibited. On the other hand, extensive training in a familiar task makes the behavior *habitual*, which is illustrated by an increase in subjects' action rate and an insensitivity to task changes (Balleine and O'Doherty, 2010), in the same manner as one could persist with the sequence of finger presses corresponding to an old pin code after this code has been recently changed. Interestingly, while healthy mammals can switch back to goal-directed behavior after a short persistence time following a task change, lesions to different brain regions can either prolong or reduce this persistence period, thus suggesting that both types of behaviors might coexist and compete for control within a modular brain architecture (Yin and Knowlton, 2006).

While goal-directed and habitual behaviors have been modeled respectively as model-based and model-free RL algorithms (Daw et al., 2005), the question of the mechanisms underlying their coordination is still an active area of research in computational neuroscience (e.g., Viejo et al., 2015; Dollé et al., 2018). Nevertheless, here we do not investigate how to operationalize this coordination and to adaptively switch from model-based to model-free control with such a bio-inspired multiple action selection system architecture, because this has been the subject of our prior work (Renaudo et al., 2014, 2015b,c). Instead, we focus here on how such an architecture enables the robot to self-monitor these action selection systems, when they are advantageous and what advantage they bring (e.g., efficiency vs. rapidity), and thus how the robot can get the ability to self-report about the way it makes decisions while learning a particular task. This ability to self-monitor can be related to the notion of self-awareness and is stated as important to allow flexible and adaptive control of a being (Van Gulick, 2017).

### 3.1. Multiple action selection systems architecture

#### 3.1.1. Overall architecture

**Figure 4:**
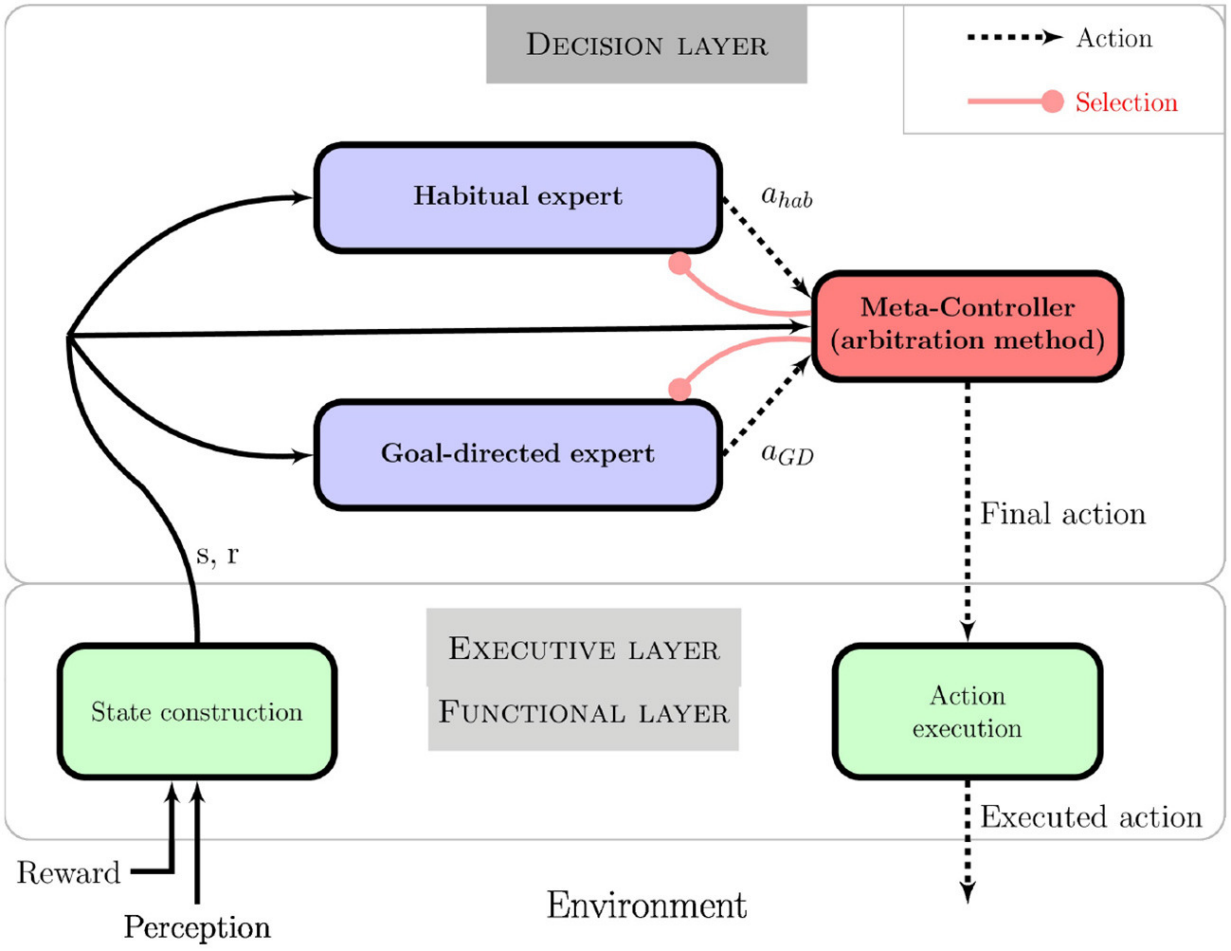
Global action selection architecture composed of two decision systems implementing corresponding behaviors: the goal-directed expert is a model-based RL algorithm whereas the habitual expert is a model-free RL algorithm. The meta-controller is in charge of monitoring different expert information, giving control to one of the two. The reward information comes from the motivational system and represents the goal of the task.

The architecture is presented in Figure 4. Each module (or *expert*) is a decision-making system that implements one way of producing actions: the goal-directed expert in a model-based RL manner and the habitual expert in a model-free RL manner. These experts learn either a model of the task or only the local state-action values based on the reward received from the motivational system and the experienced states and actions. States are received from robot sensor data processing and a set of discrete actions is made available to the action selection systems.

Whereas only one decision-making system (*expert* here) is sufficient for a robot to act autonomously, our architecture also integrates an additional component in charge of monitoring the decision-making process. The *meta-controller* analyses each expert and selects which one is actually controlling the robot at a given time step. It implements the arbitration method studied hereafter. We argue that this component is necessary to allow the robot not only to act according to the task to be fulfilled, but also to criticize and report on its own decision process.

#### 3.1.2. Possible coordination methods

In previous work (e.g., Renaudo et al., 2015b,c) we have studied and compared arbitration methods that can be separated in two categories: (i) fusion methods merging action selection probability distributions from each expert into a given state, and select an action from the final distribution and (ii) selection methods evaluating which expert is the most relevant in the current situation and let it decide about the final action. We have also defined a reference coordination method where each expert *E* among *N* experts (*N* = 2 here) has a constant and uniform probability *P*(*E*) of being selected: *P*(*E*) = 1/*N* = 0.5 in this case. This random selection has been used as a proof-of-concept in earlier work and defines the bottom performance to evaluate the interest of each particular coordination method (Renaudo et al., 2014).

Comparison of these different tested coordination methods suggests that the arbitration method should take into account multiple signals rather than only one that will miss some of the required information (Renaudo et al., 2014, 2015c). It also suggests that arbitration and expert selection should rely on information available before the experts actually compute the action to perform in the current state: this allows to save computation time of the overall decision process.

Moreover, in previous similar works (Dollé et al., 2010; Caluwaerts et al., 2012; Dollé et al., 2018), the coordination is mostly performance-based: the meta-controller in these algorithms learns which expert to recruit in each state of the task in order to maximize reward, but does not consider each expert's specific properties. Here, the habitual expert is computationally less expensive than the goal-directed expert. Thus, in case of equal performance of the experts, self-monitoring these processes should allow the meta-controller to prefer the less costly expert. On the other hand, the goal-directed expert is more efficient to update the whole policy between two interactions with the environment. When the meta-controller observes that the habitual expert proposes irrelevant actions, it can decide to select the goal-directed expert despite its high computational and time costs.

Thus, to illustrate the interest of the self-monitoring capability provided by the meta-controller, we propose a new *Learning and Cost* arbitration method described hereafter.

#### 3.1.3. A coordination method based on *learning and cost* signals

Building on these previous conclusions, we propose a new signal-based method that uses two measures of expert's status. Only the selected expert estimates the action values, which allows to save computation time and to be more reactive. One signal is directly related to this goal: the intrinsic computation cost incurred by each expert to evaluate action values. The other signal measures the experts' knowledge about the task, which can be evaluated by their learning progress.

We define ${\bar{T}}_{Hab},{\bar{T}}_{GD}$ as the mean computation times for the two experts, evaluated with exponential moving averages (see Equation 3; λ = 0.02 which is equivalent of averaging over 50 decision steps). These means are updated only when their expert has been selected to make a decision, as no cost can be measured otherwise.

(3) $$\begin{array}{r} {\bar{s}}_{t}=\left(1-\lambda \right)\;\text{·}\;{\bar{s}}_{t-1}+\lambda \;\text{·}\;s_{t} \end{array}$$

We define $\bar{\delta Q}$ as the mean variation of *Q*-values reflecting the progress of learning in the habitual expert, and $\bar{\delta P}$ as the mean variation of the transition model probabilities reflecting the progress of learning in the goal-directed expert. In model-based RL, learning is about estimating the task's transition and reward functions. Thus a measure of learning progress should refer to the model's estimation rather than the computed *Q*-values. The mean variations are updated after each action with an exponential moving average (λ = 0.2 or 5 decision steps).

In order to combine cost and learning information, we define *V*_*E*_, the *value of selecting expert E* as the weighted sums in Equation (4). We seek to preferentially select the expert that computes at the lowest cost, and that does not need to update much its knowledge because it already has enough information about how to solve the task:

(4) $$\begin{array}{r} \begin{array}{c} V_{Hab}=-\left(\alpha_{Hab}\;\text{·}\;\bar{\delta Q}+\beta_{Hab}\;\text{·}\;\bar{T_{Hab}}\right)\\ V_{GD}=-\left(\alpha_{GD}\;\text{·}\;\bar{\delta P}+\beta_{GD}\;\text{·}\;\bar{T_{GD}}\right) \end{array} \end{array}$$

The α_*i*_ and β_*i*_ parameters are the positive weights of each signal in the selection. As $\bar{\delta P}$ and $\bar{\delta Q}$ have different amplitude ranges, we set α_*GD*_ = 1 and α_*Hab*_ = 12, so the transition from goal-directed expert to habitual expert needs a strong convergence of *Q*-values in the model-free algorithm. β_*Hab*_ = β_*GD*_ = 5 in order not to bias the selection and to keep the natural difference in expert costs. Since the GD expert is computationally more costly than the Hab one, this method makes the meta-controller preferentially select the latter more often when the learning progress is equivalent between experts. These values are converted into selection probabilities *P*(*E*) using a softmax function (5) from which the selected expert is drawn. As expert *E* pays the cost of estimating actions only if it is selected, its corresponding $\bar{T_{E}}$ is only updated in the latter case.

(5) $$\begin{array}{r} P\left(E\right)=\frac{\exp\left(V_{E}/\tau \right)}{\sum_{b\in A}\exp\left(V_{E}/\tau \right)} \end{array}$$

In this method, τ is set to 1.

#### 3.1.4. Evaluation in a navigation task

**Figure 5:**
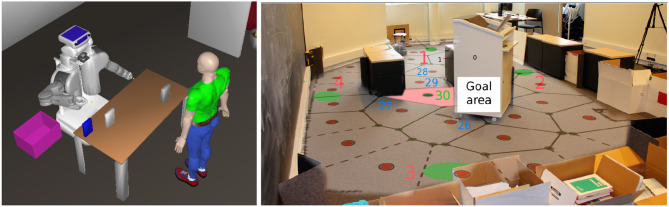
**(Left)** Setup for the Human-Robot Interaction (HRI) task from Renaudo et al. (2015a): the human and the robot collaborate to put all boxes in a trashbin. **(Right)** Arena for the navigation task. A mapping of the states produced by the robot has been manually added. The red area indicates the goal location whereas the green areas indicate starting locations of the robot. Red numbers are starting location indexes; blue numbers are some states indexes referred to later.

We evaluated the approach of combining multiple action selection systems in simulation in previous work. Especially, preliminary analyses of the reference method in a simulated human-robot interaction task (see Figure 5, left) have been reported in Renaudo et al. (2015a) and are further discussed in the next section on human-robot interaction. Here, we present novel results with the *Learning and Cost* method applied to a real robot in a navigation task.

In this task, a Kobuki Turtlebot robot has to navigate from starting locations (see Figure 5, right, green areas numbered 1–4) to the center of a 7.5 m × 3.5 m arena. Two obstacles split the arena in three corridors, the goal being located in the middle one (red area). The reward (1 unit) is given when the robot enters the goal area. It is then driven back to one of the starting locations (randomly selected). The robot localizes itself thanks to a standard particular filter based SLAM algorithm (Grisetti et al., 2007). The occupancy map built by exploring the environment is discretized into about 30 states following a regular paving. In each state, the robot can select between the 8 directions around it in the world frame. The robot controller takes care of driving the robot in the chosen direction and avoiding obstacles.We evaluate again three configurations: (i) goal-directed expert alone, (ii) habitual expert alone, (iii) both experts operate (*Combo*) and are coordinated by the meta-controller with the *Learning and Cost* method. Each configuration is evaluated 10 times, the habitual expert alone is given 2 h per repetition to learn from scratch, the goal-directed expert alone and the combination are given 1h but benefit from 1h of latent exploration (without reward in the environment) to allow the goal-directed expert to build its transition model.

### 3.2. Results

**Figure 6:**
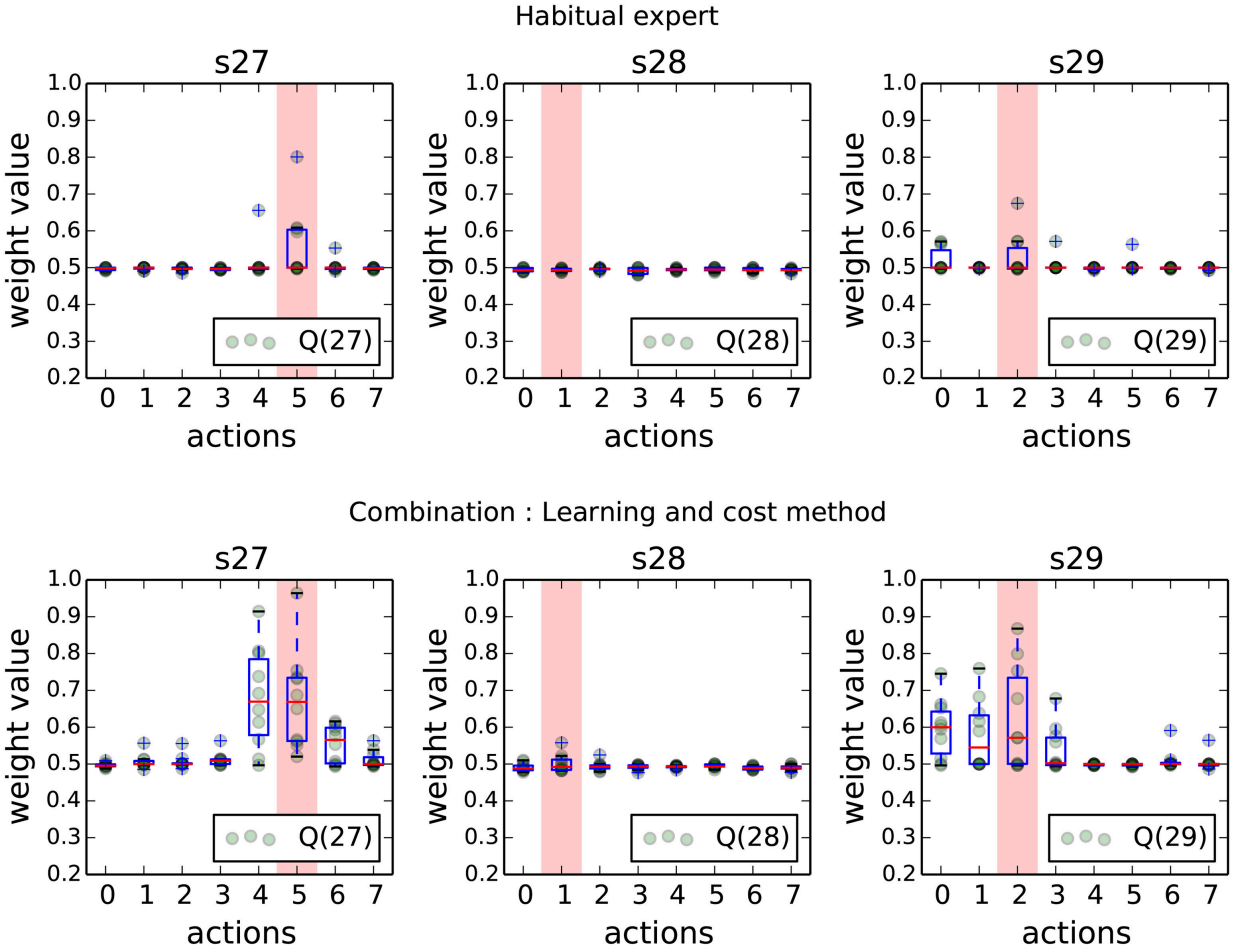
Weights of each action (direct image of *Q*-values) for the habitual expert when alone **(Top)** or combined with the goal-directed expert **(Bottom)** at the end of the navigation task. Each light green dot is the final learned value of each action. The red bar indicates the best action to take from the human perspective. These measures are shown in the states next to the goal (s27, s28, s29).

The first result of this experiment confirms the results from previous work. Figure 6 shows the final weights (which are direct images of the *Q*-values) of the habitual expert in states near the goal. When the latter is controlling the robot alone, learning is long and the *Q*-values are weakly discriminating which action will give the highest reward. When control is shared with the goal-directed expert according to the *Learning and Cost* method, the habitual expert learns faster (mostly bootstrapped through observation of the behavior produced by the GD expert), which is represented by more contrasted final values in these states.

**Figure 7:**
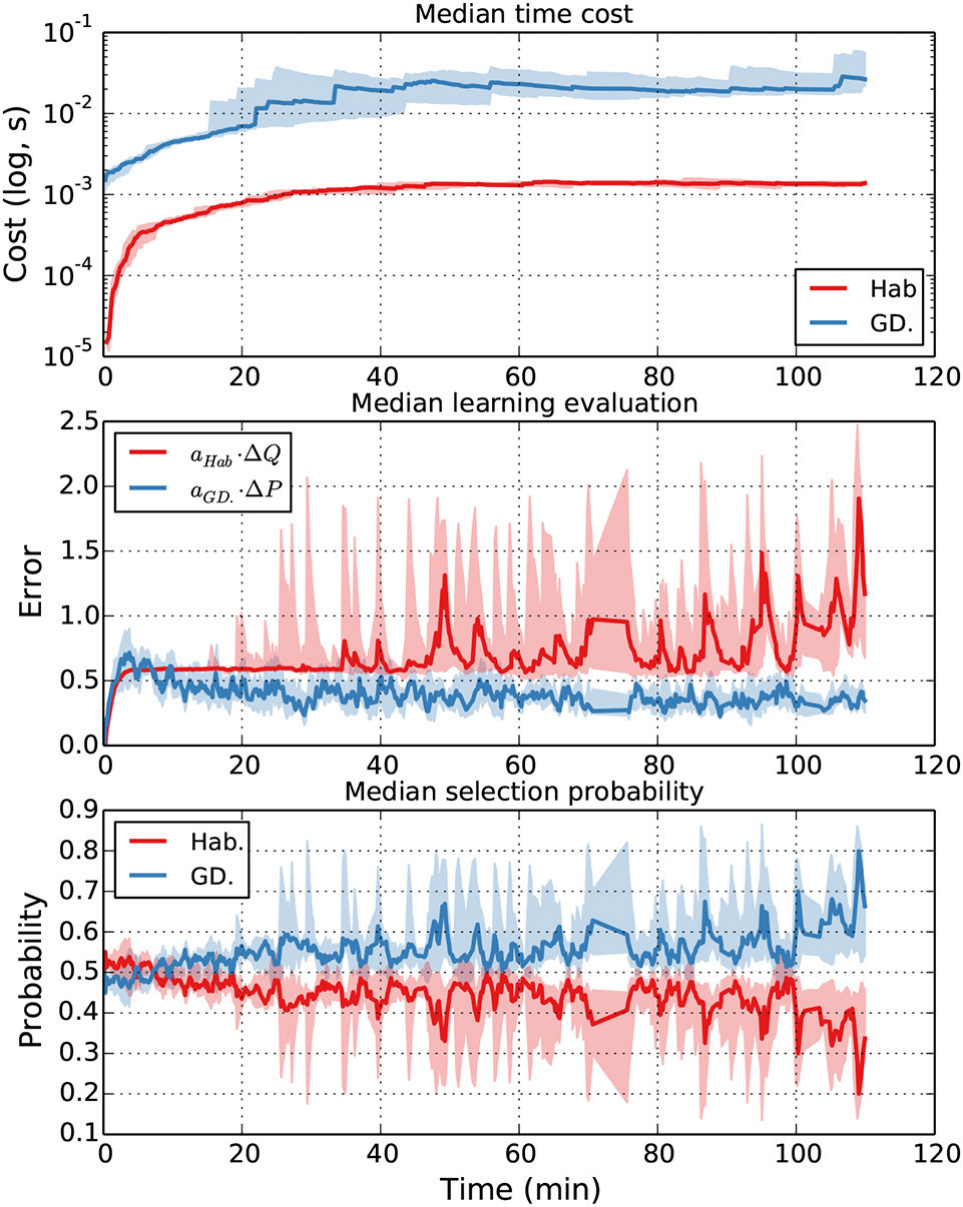
Evolution of monitored signals when both experts are controlling the robot during the navigation task. **(Top row)** shows the sliding mean cost spent by both experts for decision-making. **(Middle row)** shows the measures of learning scaled by their coefficient. **(Bottom row)** shows the evolution of the probability of selection of each expert. In these experiments, the strong parameter of the habitual expert learning measure combined with its slow convergence favors the goal-directed selection in order to reach the goal more easily (however at a high computational cost).

Figure 7 shows the monitored signals during the navigation task. Time 0 represents the initialization of a new goal location. Not surprisingly, the cost of using the goal-directed expert is one order of magnitude higher than the habitual expert cost. Interestingly, during the first minutes the habitual expert is more often selected than the GD expert until the new goal location is discovered and the GD expert starts making less error so that it gets more selected by the meta-controller. Then starts a long habit learning phase where the Hab expert slowly learns the new appropriate state-action values, which penalizes its selection (due to the high value of *a*_*Hab*_ in the criterion). As the two experts are in different states of knowledge on how to perform the task, the meta-controller mostly selects the goal-directed expert, certainly more costly but more reliable to produce the best behavior.

Here, given the real robotic setup and the natural slow learning speed of the habitual expert, the control goes mainly to the goal-directed expert. In different conditions or with longer time, the *Q*-values of the habitual expert can stabilize and this method favors its selection. Nevertheless, the important message here relative to the issue addressed in this paper is that these monitoring signals can be used by the robot to analyse its own decision-making processes and evaluate which decision-making strategies (GD or Hab) were the most efficient at different phases of the task. These capacities to monitor and report about its own performance can be integrated with representations of other agents' own abilities for efficient joint action.

## 4. Human-robot interaction: agent aware task planning

For more than a decade, the field of human-robot interaction has generated many valuable contributions of interest to the Robotics community at large. We will here give some insights concerning a particular type of interaction which is joint action, and the associated required levels of awareness. To do so, we will first explain which processes are involved in human-human joint action and then in human-robot joint action, in order to argue that minimal levels of self-awareness are required for the robot to efficiently integrate information about the effects of its actions and the effects of other agents' actions into feasible joint action plans.

### 4.1. Human-human joint action

In order to establish successful joint action, interacting agents need to be able to efficiently share and coordinate their intentions, plans, goals and actions with other participants. Put it differently, it is not enough to share a common goal between interacting agents to establish efficient joint action if each agent then individually chooses his/her own sub-goals, and simply devise his/her own individual action plan and executes it. There is a need to share a coherent joint action plan but also to coordinate actions and sub-plans between agents. This coordination is particularly crucial during the execution phase in order to ensure the successful completion of the joint action (Clark, 1996; Grosz and Kraus, 1996; Bratman, 2014; Clodic et al., 2017). One possible way to do that is from the point of view of each agent to monitor both his/her own actions and intentions as well as those of his/her partner's. Such a monitoring process can facilitate the representation and understanding of the combined impact of agents' actions on their shared goal, and the adjustment of what they do accordingly.

An important ingredient of this agent coordination process which goes in complementarity with the co-representation of tasks and actions is *joint attention*. It is an ability that has been found in apes to provide a key mechanism for establishing common ground in joint action by sharing perceptual representations of the surrounding environment and task such as the available objects and the occurring events (Tomasello and Carpenter, 2007). As an example, Brennan et al. (2008) had participants engage in a joint visual search task and showed that they were able to most of the time focus on a common space between them by directing their attention toward portions of the environment where the other was looking. Moreover, they found that their performance during such a joint search task was improved compared to the one obtained in an individual version of the task. Besides, Vesper (2014) have shown that co-agents not only engage in joint attention but also repeatedly perform transient modulations of their own movements that “reliably [have] the effect of simplifying coordination.” These are known as *coordination smoothers* and are part of a more general process called *signaling* which constitutes another phenomenon that contributes to better on-the-fly coordination. A particularly striking example is when someone exaggerates his/her own movements or reduces his/her movement variability in order to make them more easily understandable and interpretable by the other participant (Pezzulo et al., 2013).

It is important in contrast to take into account any form of joint action that may not require awareness. For instance, perception-action couplings and emerging synchronies can occur during joint action, thus making multiple individuals act in similar ways without any intention to do so, which could be viewed as a case of emergent coordination. Other processes such as *interpersonal entrainment mechanisms* can lead to emergent coordination without requiring awareness: A famous example is the one of two people sitting in rocking chairs in the same room, who sometimes unconsciously synchronize their rocking frequency (Richardson et al., 2007); Another striking example is when two people walk side by side and sometimes unconsciously synchronize their steps (van Ulzen et al., 2008). Another source of unconscious emergent coordination which is worth mentioning here is the case of *perception-action matching* (Prinz, 1997; Jeannerod, 1999; Rizzolatti and Sinigaglia, 2010). It is a situation where actions performed by a first agent and observed by a second one are considered to be mentally matched onto the second agent's own action repertoire, through the involvement of *mirror neurons* and other mental processes that enable the induction of the same action tendencies in the two agents. All these processes are thought to make agents' behavior more similar and thus more predictable, which may facilitate joint action and coordination during action execution.

Humans thus have at their disposal a vast array of processes that they can use to promote interpersonal coordination. These processes range from automatic and unintentional on-the-fly alignments and synchronizations, to sophisticated forms of reasoning and advanced representational, conceptual and communicational skills. These processes are complementary and can be combined together to enable efficient joint action. Nevertheless, for human-robot interaction, this suggests that not all joint action situations may require some degree of awareness.

### 4.2. Human-robot joint action

Human-robot joint action faces similar coordination challenges. We will explain now a way they can be translated to this case and quote some related implementation.

The robot needs to have the ability to represent itself and the human it interacts with. Doing so, it must be able to infer how each of these representations evolves along the joint action unfolding. The robot has to be able to consider perspective taking ability, knowing that representations evolve differently given each one point of view. Among others, Milliez et al. (2014) and Hiatt and Trafton (2010) endow a robot with the ability to construct a representation of other agents' mental states concerning the environment allowing it to pass the Sally and Anne test (Wimmer and Perner, 1983). Then, these mental states are used in Hiatt et al. (2011) to interpret and explain humans' behavior.

But the robot also needs to understand and take into account the effects of its own actions into the mental states of its partners, which involves a second-degree of awareness. This is done in Gray and Breazeal (2014) where the robot plays a competitive game with a human and chooses its action in order to manipulate the mental state of the human relative to the state of the world.

Each agent must also be able to asses the situation in terms of links with possible action: the objects that can be manipulated or moved, their location, the presence or absence of obstacles that could restrain some possibilities of movements. All these relate to the learned effects of actions presented in section 2 on affordances. In Sisbot et al. (2011) the robot uses the geometric information about the humans and the objects to construct symbolic knowledge as humans capabilities (an object is visible or reachable by someone), or relations between objects (an object is on/in another one). In Lemaignan et al. (2012) we have used this knowledge to anchor situated discourse during human-robot interaction. For example, if a human points at a mug saying “Give me that mug,” the robot can understand that the human wants this mug and not another one. As a corollary, joint attention appears to be also key during human-robot joint action. This is because detecting a case of joint attention permits the robot to know that whatever information it can acquire within the joint attention space can be considered as also accessible to its interactor and thus as shared knowledge. Staudte and Crocker (2011) show that people interpret robot gaze in the same way as human gaze and that a congruent use of the robot gaze helps its human partner to understand spoken references. Mutlu et al. (2013) also show that the use of speech references in congruence with robot gaze enables to disambiguate spatial references in speech, and thus to improve task performance in joint action. They also put forward that robots in general might improve task performance and the quality of user experience during human-robot collaborative interaction by using action observation.

Another capacity needed by the robot, emphasized, among others, by Tomasello et al. (2005) as a prerequisite to joint action, is to be able to read its partner's actions. Gray et al. (2005) use the concept of mirror neurons, introduced by Gallese and Goldman (1998), to infer human action goals by matching the human movement to one of its own movements (even if the robot's morphology differs from that of the human). Hawkins et al. (2014) endow a robot with the capability to probabilistically infer what the human is doing and what he will do next in order to anticipate and prepare collaborative action. This capacity relates to probabilistic transitions learned within the type of world models used for robot decision-making in section 3. Again, this suggests that duplicating world models for each agent involved in the task (Lemaignan et al., 2012) can be a good strategy for human-robot joint action. This is in line with neuroscience proposals that a substantial component of awareness resides in the development of predictive models of agency for self and others (Seth et al., 2012), and in the ability to report about these states, predictions and decisions to self and to others (Shadlen and Kiani, 2011).

Complementarily, shared task representations are important. It means, if we paraphrase Knoblich et al. (2011)'s definition, that the robot should have access to some model of what each co-agents' respective task consist in and some abilities to monitor and predict each co-agent's actions with respect to the shared goal. (Nikolaidis and Shah, 2012) present a method allowing the robot to build a shared mental model of how to perform a collaborative task by looking at human performing the task and then use it when performing the task with a human.

We have seen that both in human-human and human-robot joint attention there are similar coordination constraints that apply. However, it appears that these constraints do not necessarily apply with the same strength. For instance, when two humans interact, they both know that they share some background knowledge such as cultural information, cultural knowledge, conventions, etc. Thus they can make assumptions from both sides on what the other knows or not. In contrast, it seems much more complicated to make similar assumptions in the human-robot interaction case.

Nevertheless, we have seen that human-human joint action sometimes involves planned joint action with explicit shared goals, action plans and attentions, and sometimes involve automatic synchronization or alignment processes between partners at a more sensory-motor level. Thus one might reasonably postulate that the integration of different types of learning and decision-making within robot cognitive architectures which has previously been applied to individual robotic tasks—such as the navigation task presented in section 3 or sequential decision-making tasks in Renaudo et al. (2014)—may be relevant in the context of human-robot interaction. This could enable the robot to automatically switch between automatic/habitual behavior and planned action depending on the requirement of the task, and thus display more behavioral flexibility and efficiency during joint action with humans.

Section 3 has put forward the hypothesis that the same coordination mechanisms for model-based and model-free reinforcement learning within robot architecture could be relevant both for non-social and social tasks in the context of the human-robot interaction task proposed by Alami (2013) and Lemaignan et al. (2017). Nevertheless, in this previous section the robot only achieved individual action plans, not joint action plans.

**Figure 8:**
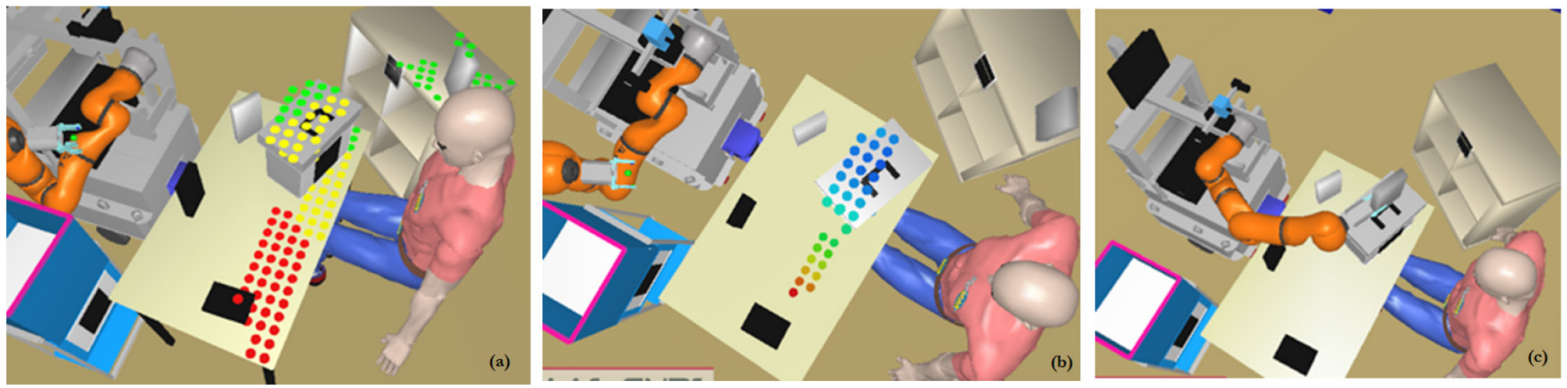
Task of making an object accessible by the human to the robot (Pandey et al., 2013): **(a)** Places on the support planes where the human can put something with least effort. **(b)** Weighted points where the robot can support the human by taking the object. **(c)** The planner found a possible placement of the object on the box from where it is feasible for the robot to take. Note that, because of the object-closeness based weight assignment, this placement also reduces the human's effort to carry the object.

A more general illustration of awareness of each agent's actions' task-dependent effects and abilities that is required for joint action plans is shown in Figure 8. Again here, human and robot have to cooperate by putting some objects in certain placements where some are accessible only to the human or the robot. The robot has to elaborate a representation of different sub-spaces on the table so that it understands that some objects or places are accessible to the human. The robot tries to estimate visibility and reachability of the human and of itself (Pandey et al., 2013; Pandey and Alami, 2014) in order to determine the right places to use and where they can exchange objects. Also, the robot here has the capability to estimate the effort of the human in order to select the most pertinent places.

However, there is still a gap between such representations and those are required for the execution of an effective shared action plan. Indeed the robot should be able not only to compute the perspective of its human partner and use it to estimate how he can assess the current situation but also to estimate his current knowledge of the state of the task and the corresponding shared plan.

In Devin and Alami (2016) and Devin et al. (2017) we have developed, within the architecture described in Lemaignan et al. (2017), a framework that permits a robot to estimate the mental state of its human partner with respect to a given collaborative task achievement. We have moreover proposed a form of mental states which contains several task-relevant informations such as the states of the world, of the goals, actions and plans. To do so, the robot has to estimate and to permanently update the spatial perspective of its partners. It moreover has to constantly track their activity. Once these mental states representations are constructed and handled by the robot, it can use them to perform joint actions with humans. In the context of the present project, we have mostly investigated this in cases of collaborative objects manipulation. An advantage of the approach is to permit the robot to adapt online to the human's behavior and intention changes, while at the same time informing the human when needed in a non-intrusive manner, for instance by avoiding to give unnecessary information that the human could infer himself through observation or through deduction from past events.

**Figure 9:**
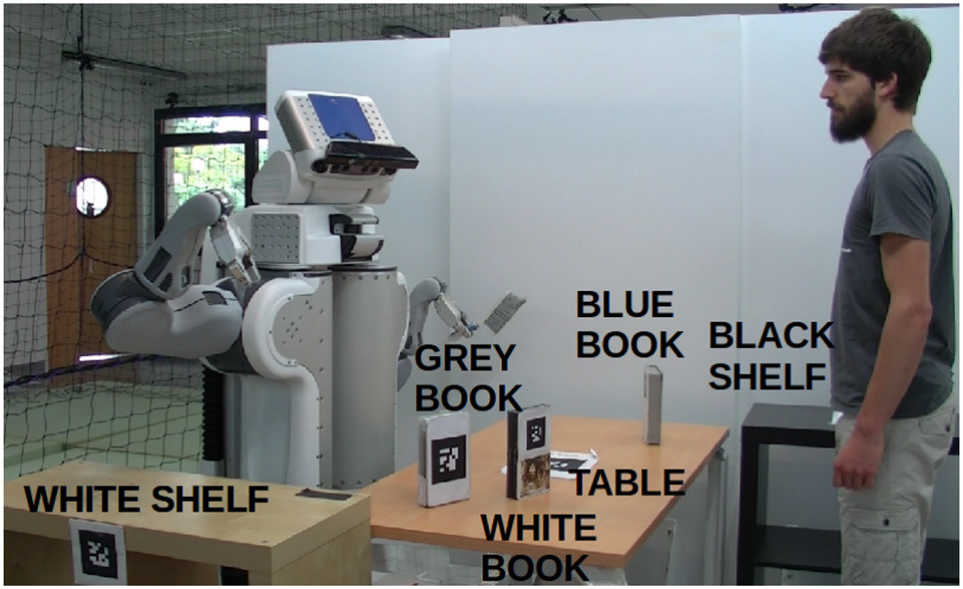
Initial state of the world in the Clean the table scenario. In this task, the robot and the human share the goal of cleaning the table together.

**Figure 10:**
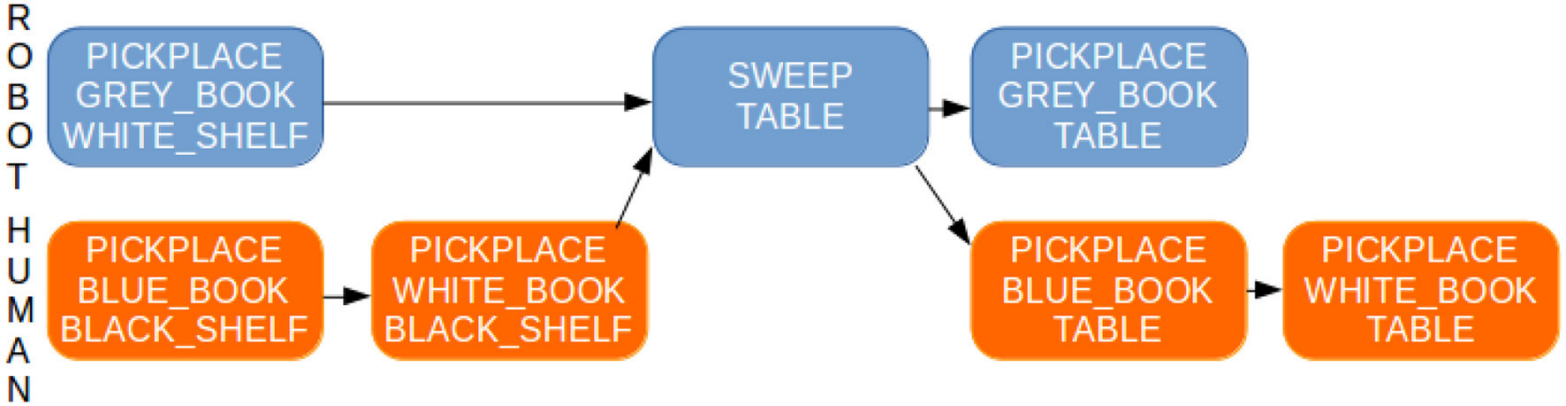
Shared plan computed by the robot to solve the joint goal: first removing the three objects (books) that are located on the table, then sweeping the table in order to clean it and finally placing the objects back on the table. While cooperatively achieving the task, the robot will be able to detect and assess correctly why the human partner stays idle, for instance in cases where, due to a momentary absence, the human may have missed the fact that robot has swept the table.

As an illustration, let us consider a PR2 robot sharing with a human the goal of cleaning a table, that is, to first remove all objects on the table, then to sweep it, and afterwards to replace all objects back on the table. Figure 9 shows the initial state of the world. On the table there is a blue book which is only reachable by the human, a gray book accessible only by the robot, and a white book reachable by both. Two actions are available to the robot: *pick-and-place* and *sweep*. The former can be executed by the robot only when the considered object and support on which to place the object are reachable by the robot. The latter can be executed on a surface only when it is again reachable by the robot and when there are not any objects on it. Figure 10 illustrates the initial plan produced by the robot to achieve the goal.

The robot, equipped with such enlarged awareness ability, is not able to perform joint tasks more fluently. to reduce unnecessary communication and to choose the most pertinent way to inform about the state of the plan, to produce a less intrusive behavior of the robot but also potentially detect situations where human lacks an information allowing him to act and also the robot can in certain cases prevent human mistakes due to a wrong evaluation of the current state of the task.

These contributions involved pre-defined world, task and human models so that the robot can plan complex action plans involving collaborative human-robot task achievement with a human-aware task planner (HATP) (Alami et al., 2011; Lallement et al., 2014) and the associated high-level robot controller (Devin and Alami, 2016; Devin et al., 2017). This however did not involve a learning process. We have proposed in section 3 an extension of this work by considering that the subparts of the action sequence that are repeatedly performed by the robot in the same manner in this condition can be learned by the model-free habit learning system of their architecture. This is similar to habits learned by humans in conditions where repetitive behaviors are always occurring in the same context and in the same manner. This could enable the robot to autonomously detect and thus be aware of which situations are stable enough and repetitive enough to avoid systematically using the slow and costly action planning system. In addition, this framework should also enable the robot to automatically detect when environmental changes require to break the habit and switch back to the planning of new action sequences. Nevertheless, an extension of this work which is still under investigation consist in extending this framework by also enabling the robot to represent a world model associated to the human's actions' effects. This should permit to use model-based reinforcement learning to refine the world, the task and the human models used by HATP and the robot supervision system in order to find other action plans that could not be anticipated by the human experimenter. This could also lead to further awareness by the robot of which joint action plans are predictable by the human, and which should appear as new.

## 5. Self-aware decision making

### 5.1. General approach

Planning in the field of AI is usually considered as the problem of building a sequence of actions selected from a predefined set in order to achieve a goal specified by a user or an external system (see Khamassi et al., 2016; Ingrand and Ghallab, 2017 for recent reviews). Classical planning is mostly based on First-order Predicate Logic or extensions thereof. If there are uncertainties on states, or on action outcomes, a probabilistic formulation is used and MDPs/POMDPs are the main tools.

The question addressed here is how can a system decide for its *own* goals, without being requested by an external agent? How can it decide to change goals dynamically? These questions are important because their answers determine if the agent is capable of a form of volition. Addressing them has lead to design a system capable of meta-reasoning to reflect on its objectives and on the way it is accomplishing them. In other words, the system described next is reasoning on its own motivations and actions, a feature we believe is related to self-awareness.

We want to build a system able to reach potentially concurrent goals and to manage resources such as energy and time, in an uncertain dynamic world. We aim for autonomous initiative and decision-making, so that the agent does not only react to particular stimuli or direct external requests, but most importantly selects by itself goals to achieve.

We consider the notion of *motivation* as the basis for bootstrapping the system's behavior, the trigger for a capacity of taking initiatives. The question of internal motivations has often been overlooked in the autonomous robotics literature: motivations are usually identified as simple drives emerging from external stimuli, whose dynamics are entirely dictated by the metabolism (e.g., decreasing energy level) and the occasional unconditional rewarding signals issued from the environment (such as locations for energy charging). The resulting systems are thus not purely reactive, but they can neither be considered as deliberative and motivationally autonomous because they lack an evaluation and selection among motivations. The selection is rather usually based on inhibitory signals resulting from external stimuli, such as in the multiple implementations of the subsumption architecture (Brooks, 1986).

Here, we want to investigate the potential advantage that an artificial system could have in developing its own preferences, i.e., to associate virtual rewards (to be distinguished from reward predictions used in actor-critic models, for example) to specific states which seem to have a key role in obtaining long-term rewards and should thus become intrinsically rewarding. These virtual rewards would be created by the motivational system, while the learning systems would remain unaware of the real or virtual aspect of the rewards they are manipulating. A possible advantage could be to set key-points where a reset of the reward discount mechanisms would be made, thus avoiding the problem of the discounted reward vanishing when trying to learn to reach very long-term goals.

This could account for example for the behavior of rats in the task studied in, where the stimulus seems to become a reward in itself, even when the food is not consumed. These virtual rewards could then be used for learning by model-based and model-free systems, as has been proposed by Lesaint et al. (2014) to account for these rats' behavior in a similar manner to the one presented in section 3. Virtual rewards could also explain how getting more money, a normally intermediate step which can indirectly lead to unconditional rewards like food, can become a reward in itself.

We focus here on the higher level of the robot cognitive architecture and propose to transform it into a deliberation system involving a self-awareness capacity. For this we hypothesize two layers of decision-making: (i) a higher level one called *deliberation* layer for solving multiple goal situations given motivations (using an “intentional module," context and long-term objectives, producing a “goal agenda" as input to (ii) a lower level goal-oriented planning system called the *operational* layer which will decide of the more precise course of actions to achieve the goals. This planning system is associated with a supervisory control system, which enables to control action execution as in classical systems.

The notion of *motivation* proposed in this paper, is a structure consisting of (individual or chained) goals, which may be permanently active or not, and to which we associate rewards. We aim to predict the precise effects of the resolution of a goal on the world and on other motivations, in order to compute a high-level plan, employing goal-reaching *policies* in the same way that we usually use *actions* in an MDP.

We hence develop an architecture that:

1. handles motivations,
2. computes possible policies for each motivation,
3. predicts the behavior of each policy and its effect on motivations,
4. predicts the effects of a chain of policies,
5. finds an optimal arrangement of these policies, maximizing the sum of the rewards obtained by the related motivations for a given time-horizon.

### 5.2. Motivations

Motivations are modeled as finite state machines corresponding to specific objectives, which can be permanent and basic, such as “maintain a high battery level," or complex and chained, such as “activate device A and then device B," etc.

The state of a motivation changes when there is a relevant change in the state of the world. To check if the conditions are met for changing the motivation state, an observation of world state transitions (*ws, a, ws*′) is required, where *ws* is the initial world state, *a* is the executed action, and *ws*′ is the resulting world state. World state transitions provide information that can trigger *motivation transitions*, i.e., changes in motivation states from *ms* to *ms*′. A *motivation transition* can be defined as an expression: (*ms*, (*ws, a, ws*′)) → *ms*′. We associate a positive or negative *reward r* to each motivation transition, reflecting its importance.

A rewarded motivation transition *rmt* starting from *ms*1 and leading to *ms*2 is called an *available-rmt* when the current motivation state is *ms*1. It *becomes activated* (or *triggered*) when the corresponding world transition (*ws, a, ws*′) happens, changing the motivation state to *ms*2 and obtaining the corresponding positive or negative reward (*r*). The maximization of the sum of these rewards will be sought by the deliberation system.

### 5.3. Decision system

**Figure 11:**
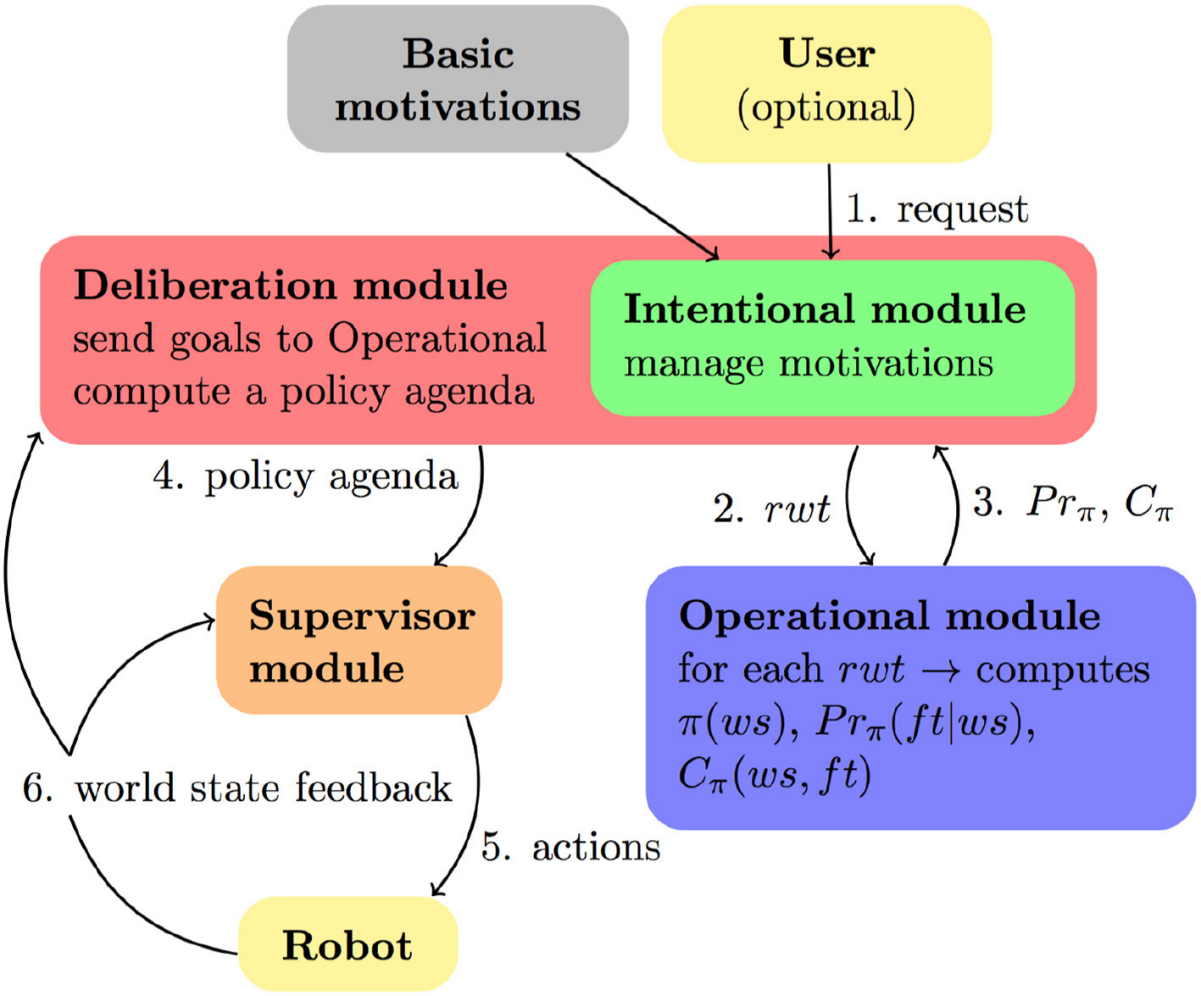
Decision-making architecture including operational, intentional and deliberation modules. The deliberation module implements a meta-reasoning capability.

The architecture of the decision-making system is organized into the following modules (Figure 11):

- An *intentional module*, which manages the agent's objectives in the form of motivations. It is embedded in the deliberation modules (see next). It creates a list of motivations *msv*, containing the current states of all motivations. Consequently, given a *msv*, it is possible to know all the active rewarded motivation transitions originating from those current states, called *available-rmts*. This module is also responsible for keeping motivations up-to-date, depending on the world state evolution.
- An *operational module*, which computes policies based on the motivations automata, and computes predictions on resulting policies. It's based on an MDP.
- A *deliberation module*. Its role is to provide to the operational module rewarded word transitions *rwt* to reach, to enable it to build predictable solutions that will trigger the corresponding rewarded motivation transitions *rmt*. The deliberation module then computes the effects of these policies on the world state and on all motivations. These policies are used as macro-actions to compute a conditional high-level plan for maximizing the sum of the motivation rewards. This plan is called *policy agenda* handed to the supervisory system for execution. Thus, this module actually reasons on the active motivations, and on the best way to satisfy them using the policies the operation module can offer to achieve them. In other words, this is a meta-reasoning capacity, which we believe a core feature of self-awareness. The robot is not simply driven by its motivations, neither by a classical planning ability which determines a course of actions to achieve a goal. The robot determines its own goals by pondering how to satisfy its motivations and based on its planning results.

In summary, the actions are based on motivations that are driving the system's decisions. Motivations trigger the computation of policies to achieve them. Deliberation evaluates the policies to select the more rewarding actions. This achieves a meta reasoning capacity.

## 6. Cognitive architecture

**Figure 12:**
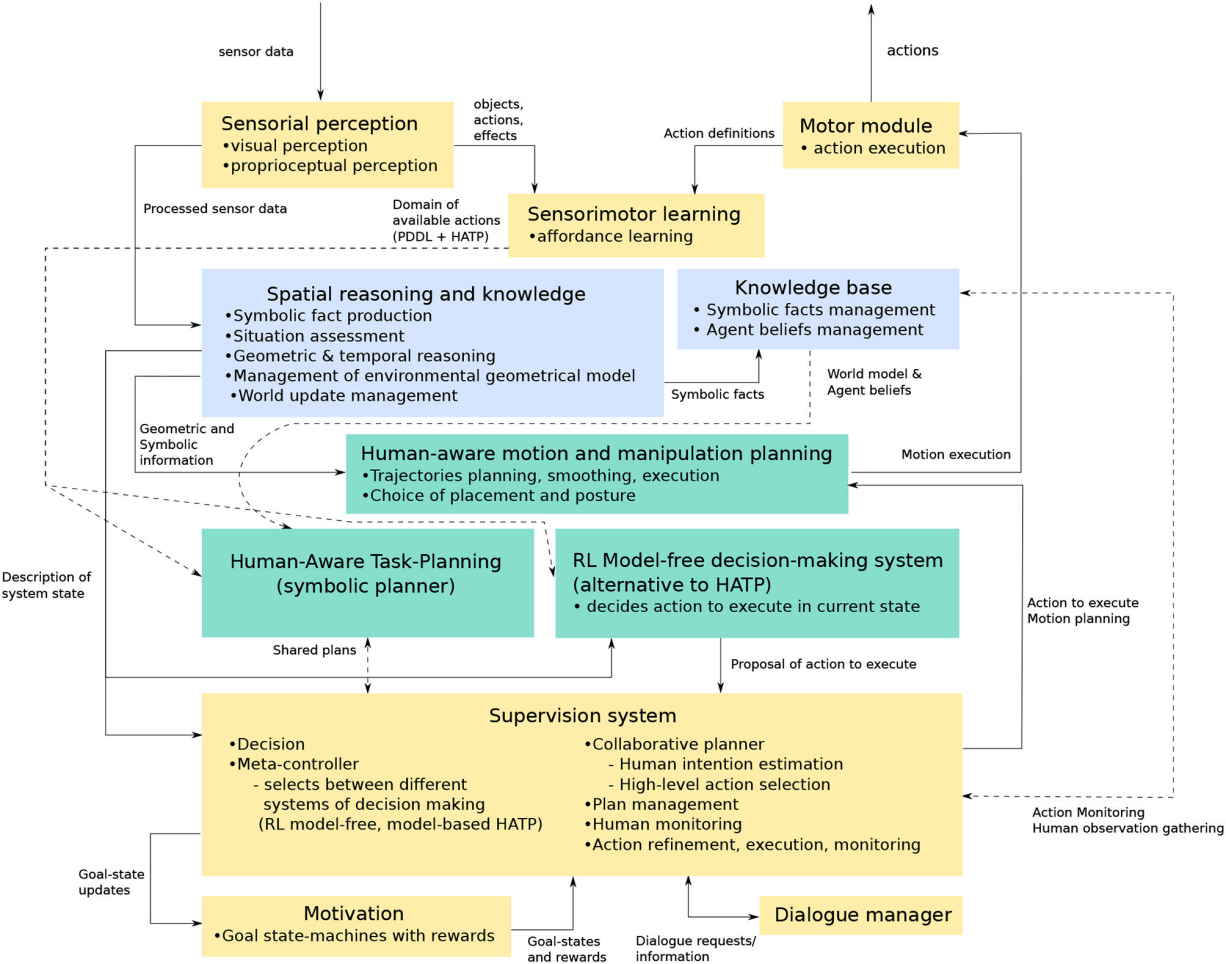
The global cognitive architecture employed in the RoboErgoSum project. Blue modules are responsible for generating and managing the symbolic knowledge. Decision-making modules are shown in green. Solid and dashed lines are used only to improve diagram readability where lines cross, and are otherwise identical in meaning.

The RoboErgoSum project employs a cognitive architecture designed for providing a robot with the necessary skills for autonomous activity in an unknown environment. The software architecture of the project is shown in Figure 12. Although we present an architecture unifying the modules detailed in the previous sections, a validation of the global architecture is yet to be done. Nevertheless, parts of this architecture were validated separately, as detailed at the end of this section.

The architecture contains modules for:

- sensing and acting in the environment (Sensorial perception and Motor modules),
- sensorimotor learning (sensorimotor learning module),
- symbolic knowledge generation and management (blue modules: Spatial reasoning and knowledge, Knowledge base)
- decision and action planning (green modules: Human-aware task planning, Reinforcement Learning model-free decision making system, Human-aware motion and manipulation planning),
- controlling the modules (Supervision system),
- goal management (Motivation module),
- dialogue management.

The interconnections between the modules are structured as follows.

The **Sensorial perception module** contains the innate set of perceptual abilities for perceiving the environment (visual perception and proprioception). The **Motor module** contains the innate set of action primitives available to the robot, which allow it to interact with the environment.

The **Sensorimotor learning** module processes the available pre-processed inputs (i.e., objects detected, actions performed, measured effects) to discover and learn which interactions are available to the robot in the current environment (i.e., affordance learning). It also generates the set of available actions that were learned after the interaction with the environment, together with their pre-conditions and post-conditions.

The **Spatial reasoning and knowledge** and the **Knowledge base** modules (Lemaignan et al., 2017) generate and store symbolic data about the perceived environment. This data is then used in the action planning phase by the corresponding modules: **Human-aware task planning module** (Lallement et al., 2014), and the **Human-aware motion and manipulation planning module** (Sisbot et al., 2007; Sisbot and Alami, 2012). Knowledge about the current state and the available actions is used by the **Reinforcement Learning model-free decision making system**.

The **Supervision system** communicates with the aforementioned modules to decide which action planning system to employ, to perform on-line plan correction, and to monitor the activity of humans with which it interacts.

The **Motivation** module manages the set of goals that have to be achieved by the robot. Together with the action planning modules, it computes the optimal set of actions to perform, so as to obtain the highest reward in the given time horizon.

We validated several pieces of this architecture, using different sets of modules. We employed in an affordance-learning context the combination of modules responsible for Sensorial perception, Motor action execution, and Sensorimotor learning (the 3 yellow modules on the top of the Figure 12), previously described in section 2 (Chavez-Garcia et al., 2016b,a). Similarly, in a human-robot collaboration setting, we employed the modules for Sensorial perception, Motor action execution, Spatial reasoning and knowledge, Knowledge base, Supervision system, Human-aware task planning, Human-aware motion and manipulation planning, Motivation, and Dialogue Manager (Alami, 2013; Devin and Alami, 2016; Lemaignan et al., 2017). We also linked these modules with a Reinforcement-Learning model-free decision making system (Renaudo et al., 2015a), as described in section 3.

In spite of these advancements, a validation of the global architecture remains to be done. This would require a considerable engineering effort for integrating the presented modules, as not all the interfaces between them are present today.

## 7. Lessons learned and conclusion

Affordance learning mechanisms presented in section 2 to learn effects of actions constitute a first level of awareness of the distinction between self, other agents, movable objects and fixed elements of the environment. The learned action effects can moreover be used as transition estimates between states of the environment which can be used as world models for other learning and decision-making components of the robot cognitive architecture.

A second level of awareness can be permitted by having the agent monitor various dynamic signals about the environment and its performance to decide which learning strategy is relevant at any given moment, between the model-based and model-free strategies presented in section 3. This not only provides more behavioral flexibility and decisional autonomy, as we have previously argued (Renaudo et al., 2014; Khamassi et al., 2016), but as we proposed here can constitute a way for the robot to further evaluate and report about how it learned a task, which strategies were efficient in particular circumstances. Further investigations in this direction should study whether this enable more generalization for the robot when it can recognize similar circumstances (measured through the same performance and task monitoring signals) in which it could attempt similar learning strategies successfully. A further progress in integration could permit these monitoring mechanisms to inform in return the affordance learning module to enrich the list of effects associated to actions with long-term effects in terms of different task resolutions. While this is still an ongoing part of the present project and requires further exploration, we argue here that such an integration of robot cognitive abilities should permit wider and long-term-oriented awareness of the agent to mentally represent what the tasks it can and cannot do with regards to its current capacities and past experience.

Besides, a particularly interesting lesson that we have learned from studying robot learning mechanisms in social and non-social tasks (section 4) is the observation that similar coordinations mechanisms of model-based and model-free learning strategies with a meta-controller can be relevant in both contexts. As the review of the human-human joint action literature suggests, joint action also involves both conscious model-based joint intention and unconscious action synchronization. Both are nevertheless important to enable intentional and unconscious signaling which enable each agent to be more predictable (and thus *readable*) by her coactor for efficient joint action. Application of the coordination of model-based and model-free learning mechanisms to human-robot interaction that we have initiated suggests that it could also permit the robot to become aware of which tasks performed in interaction with the human can be performed habitually, and which require a constant monitoring and reevaluation of possible action consequences through learned world models. This can further promote the development of internal models of what the human can and cannot do, which objects it can or cannot reach, as well as models of what the respective tasks of each of the co-agents.

Interestingly, some of the previous human-robot joint action experiments that we have previously done and summarized here suggest that a simple duplication of the robot's individual learning mechanisms presented here could be done within the robot's internal representations for each agent involved in the task. In other words, world models can be learned and generalized for each agent involved in the task (Lemaignan et al., 2012) in order to endow a robot with the capability to probabilistically infer what the human is doing and what he will do next in order to anticipate and prepare collaborative action. This is in line with neuroscience proposals that a substantial component of awareness resides in the development of predictive models of agency for self and others (Seth et al., 2012), and in the ability to report about these states, predictions and decisions to self and to others (Shadlen and Kiani, 2011).

Section 5 presented progress in the development of further awareness abilities, this time about the agent's decisions on its goals and motivations represented by finite state machines. We presented a system for managing multiple concurrent and permanent objectives, performing probabilistic reasoning with MDPs and capable of reasoning its plans to decide for the most rewarding actions. The deliberative system has a modular architecture, which separates the planning from the goal-managing entity, allowing for an easy integration into an existing robotic cognitive architecture.

Finally, we presented a global cognitive architecture designed to permit the integration of these different cognitive functions.

The whole work reported in this paper provides insights about how to achieve a self-aware system and to decipher what is awareness and what it is not, by monitoring the processes of the robot and recognizing when they solved a task with explicit deliberation and model-based strategies, or through unconscious model-free learning. It would be interesting to be able to measure the amount of integrated information in the robot cognitive architecture during these different processes, and see whether we can differentiate the two quantitatively, in agreement with the integrated information theory of Oizumi et al. (2014). This would need to be the subject of future research projects.

## Author contributions

RA, RC, AC, BG, and MK designed the research. RC is the project leader. MA, R-OC-G, SD, RG, PL-V, and ER made developments and performed the experiments. All authors contributed to data analyses and interpretation. RA, MA, RC, R-OC-G, AC, SD, BG, RG, MK, PL-V, and ER wrote the manuscript.

### Conflict of interest statement

The authors declare that the research was conducted in the absence of any commercial or financial relationships that could be construed as a potential conflict of interest.

## References

[B1] Alami R. (2013). On human models for collaborative robots, in 2013 International Conference on Collaboration Technologies and Systems, CTS 2013, May 20-24, 2013 (San Diego, CA), 191–194. 10.1109/CTS.2013.6567228

[B2] Alami R. Warnier M. Guitton J. Lemaignan S. Sisbot E. A. (2011). When the robot considers the human…, in Proceedings of the 15th International Symposium on Robotics Research (Flagstaff, AZ).

[B3] Anderson J. R. Bothell D. Byrne M. D. Douglas S. Lebiere C. Qin Y. (2004). An integrated theory of the mind. Psychol. Rev. 111, 1036–1060. 10.1037/0033-295X.111.4.1036 15482072

[B4] Andries M. Chavez-Garcia R. O. Chatila R. Giusti A. Gambardella L. M. (2018). Affordance equivalences in robotics: a formalism. Front. Neurorobot. 12:26. 10.3389/fnbot.2018.00026 29937724 PMC6002533

[B5] Balleine B. W. O'Doherty J. P. (2010). Human and rodent homologies in action control: corticostriatal determinants of goal-directed and habitual action. Neuropsychopharmacology 35:48. 10.1038/npp.2009.131 19776734 PMC3055420

[B6] Bratman M. E. (2014). Shared Agency: A Planning Theory of Acting Together. New York, NY: Oxford University Press.

[B7] Brennan S. E. Chen X. Dickinson C. A. Neider M. B. Zelinsky G. J. (2008). Coordinating cognition: the costs and benefits of shared gaze during collaborative search. Cognition 106, 1465–1477. 10.1016/j.cognition.2007.05.012 17617394

[B8] Brooks R. A. (1986). A robust layered control system for a mobile robot. IEEE J. Robot. Automat. 2, 14–23. 10.1109/JRA.1986.1087032

[B9] Caluwaerts K. Staffa M. N'Guyen S. Grand C. Dollé L. Favre-Félix A.. (2012). A biologically inspired meta-control navigation system for the psikharpax rat robot. Bioinspir. Biomimet. 7:025009. 10.1088/1748-3182/7/2/025009 22617382

[B10] Chavez-Garcia R. O. Andries M. Luce-Vayrac P. Chatila R. (2016a). Discovering and manipulating affordances, in International Symposium on Experimental Robotics (ISER) (Tokyo).

[B11] Chavez-Garcia R. O. Luce-Vayrac P. Chatila R. (2016b). Discovering affordances through perception and manipulation, in IEEE/RSJ International Conference on Intelligent Robots and Systems (IROS) (Daejeon).

[B12] Chella A. Manzotti R. (eds). (2007). Artificial Consciousness. Exeter: Imprint Academic.

[B13] Clark H. H. (1996). Using language. Cambridge: Cambridge University Press.

[B14] Clodic A. Pacherie E. Alami R. Chatila R. (2017). Key Elements for Human-Robot Joint Action. Cham: Springer International Publishing.

[B15] Comaniciu D. Meer P. Member S. (2002). Mean shift: a robust approach toward feature space analysis. Patt. Anal. Mach. Intell. IEEE Trans. 24, 603–619. 10.1109/34.1000236

[B16] Daw N. D. Niv Y. Dayan P. (2005). Uncertainty-based competition between prefrontal and dorsolateral striatal systems for behavioral control. Nat. Neurosci. 8, 1704–1711. 10.1038/nn1560 16286932

[B17] Dayan P. (2009). Goal-directed control and its antipodes. Neural Netw. 22, 213–219. 10.1016/j.neunet.2009.03.004 19362448

[B18] Dehaene S. Naccache L. (2001). Towards a cognitive neuroscience of consciousness: basic evidence and a workspace framework. Cognition 79, 1–37. 10.1016/S0010-0277(00)00123-2 11164022

[B19] Devin S. Alami R. (2016). An implemented theory of mind to improve human-robot shared plans execution, in The Eleventh ACM/IEEE International Conference on Human Robot Interation, HRI 2016, March 7-10, 2016 (Christchurch), 319–326.

[B20] Devin S. Clodic A. Alami R. (2017). About decisions during human-robot shared plan achievement: who should act and how?, in Social Robotics - 9th International Conference, ICSR 2017, November 22-24, 2017, Proceedings (Tsukuba), 453–463.

[B21] Dollé L. Chavarriaga R. Guillot A. Khamassi M. (2018). Interactions of spatial strategies producing generalization gradient and blocking: a computational approach. PLoS Comput. Biol. 14:e1006092. 10.1371/journal.pcbi.1006092 29630600 PMC5908205

[B22] Dollé L. Sheynikhovich D. Girard B. Chavarriaga R. Guillot A. (2010). Path planning versus cue responding: a bioinspired model of switching between navigation strategies. Biol. Cybern. 103, 299–317. 10.1007/s00422-010-0400-z 20617443

[B23] Fikes R. E. Nilsson N. J. (1971). Strips: a new approach to the application of theorem proving to problem solving, in Proceedings of the 2Nd International Joint Conference on Artificial Intelligence, IJCAI'71 (San Francisco, CA: Morgan Kaufmann Publishers Inc.), 608–620.

[B24] Flagel S. B. Clark J. J. Robinson T. E. Mayo L. Czuj A. Willuhn I. (2011). A selective role for dopamine in stimulus-reward learning. Nature 469, 53–57. 10.1038/nature09588 21150898 PMC3058375

[B25] Gallese V. Goldman A. (1998). Mirror neurons and the simulation theory of mind-reading. Trends Cogn. Sci. 2, 493–501. 10.1016/S1364-6613(98)01262-5 21227300

[B26] Gibson J. (1977). The theory of affordances, in Perceiving, Acting, and Knowing: Toward an Ecological Psychology, eds Shaw R. E. Bransford J. (Hillsdale, NJ: Lawrence Erlbaum Associates), 67–82.

[B27] Gray J. Breazeal C. (2014). Manipulating mental states through physical action. Int. J. Soc. Robot. 6, 315–327. 10.1007/s12369-014-0234-2

[B28] Gray J. Breazeal C. Berlin M. Brooks A. Lieberman J. (2005). Action parsing and goal inference using self as simulator, in Robot and Human Interactive Communication, 2005. ROMAN 2005. IEEE International Workshop on (Nashville, TN: IEEE), 202–209.

[B29] Grisetti G. Stachniss C. Burgard W. (2007). Improved techniques for grid mapping with rao-blackwellized particle filters. IEEE Trans. Rob. 23, 34–46. 10.1109/TRO.2006.889486

[B30] Grosz B. J. Kraus S. (1996). Collaborative plans for complex group action. Artif. Intell. 86, 269–357. 10.1016/0004-3702(95)00103-4

[B31] Haggard P. Tsakiris M. (2009). The experience of agency: feelings, judgments, and responsibility. Curr. Direct. Psychol. Sci. 18, 242–246. 10.1111/j.1467-8721.2009.01644.x

[B32] Hawkins K. P. Bansal S. Vo N. N. Bobick A. F. (2014). Anticipating human actions for collaboration in the presence of task and sensor uncertainty, in Robotics and automation (ICRA), 2014 ieee international conference on (Hong Kong: IEEE), 2215–2222.

[B33] Hiatt L. M. Harrison A. M. Trafton J. G. (2011). Accommodating human variability in human-robot teams through theory of mind, in IJCAI Proceedings-International Joint Conference on Artificial Intelligence, Vol. 22 (Barcelona), 2066.

[B34] Hiatt L. M. Trafton J. G. (2010). A cognitive model of theory of mind, in Proceedings of the 10th International Conference on Cognitive Modeling (Philadelphia, PA: Drexel University), 91–96.

[B35] Hood B. (2012). The Self Illusion: How the Social Brain Creates Identity. Oxford, UK: Oxford University Press.

[B36] Ingrand F. Ghallab M. (2017). Deliberation for autonomous robots: a survey. Artif. Intell. 247, 10–44. 10.1016/j.artint.2014.11.003

[B37] Jeannerod M. (1999). The 25th bartlett lecture. Q. J. Exp. Psychol. Sect. A 52, 1–29. 10.1080/713755803 10101973

[B38] Khamassi M. Girard B. Clodic A. Sandra D. Renaudo E. Pacherie E. (2016). Integration of action, joint action and learning in robot cognitive architectures. Intell. Assoc. Pour Recher. Sci. Cogn. 2016, 169–203. Available online at: http://intellectica.org/fr/integration-de-l-action-de-l-action-conjointe

[B39] Knoblich G. Butterfill S. Sebanz N. (2011). Psychological research on joint action: theory and data. Psychol. Learn. Motivat. Adv. Res. Theory 54:59 10.1016/B978-0-12-385527-5.00003-6

[B40] Kober J. Bagnell D. Peters J. (2013). Reinforcement learning in robotics: a survey. IJRR J. 11, 1238–1274. 10.1177/0278364913495721

[B41] Koch C. Massimini M. Boly M. GT. (2016). Neural correlates of consciousness: progress and problems. Nat. Rev. Neurosci. 17, 307–321. 10.1038/nrn.2016.22 27094080

[B42] Lallement R. de Silva L. Alami R. (2014). HATP: an HTN planner for robotics. CoRR abs/1405.5345.

[B43] Lehman J. F. Laird J. Rosenbloom P. (2006). A Gentle Introduction to Soar : An Architecture for Human Cognition: 2006 Update. Available online at: http://ai.eecs.umich.edu/soar/sitemaker/docs/misc/GentleIntroduction-2006.pdf

[B44] Lemaignan S. Ros R. Sisbot E. A. Alami R. Beetz M. (2012). Grounding the interaction: anchoring situated discourse in everyday human-robot interaction. Int. J. Soc. Robot. 4, 181–199. 10.1007/s12369-011-0123-x

[B45] Lemaignan S. Warnier M. Sisbot E. A. Clodic A. Alami R. (2017). Artificial cognition for social human-robot interaction: an implementation. Artif. Intell. 247, 45–69. 10.1016/j.artint.2016.07.002

[B46] Lesaint F. Sigaud O. Flagel S. B. Robinson T. E. Khamassi M. (2014). Modelling individual differences in the form of pavlovian conditioned approach responses: a dual learning systems approach with factored representations. PLoS Comput. Biol. 10:e1003466. 10.1371/journal.pcbi.1003466 24550719 PMC3923662

[B47] Lewis P. R. Chandra A. Parsons S. Robinson E. Glette K. Bahsoon R. (2011). A survey of self-awareness and its application in computing systems, in Self-Adaptive and Self-Organizing Systems Workshops (SASOW), 2011 Fifth IEEE Conference on (IEEE), 102–107.

[B48] Marks-Tarlow T. (1999). The self as a dynamical system. Nonlin. Dyn. Psychol. Life Sci. 3, 311–345. 10.1023/A:1021958829905

[B49] Milliez G. Warnier M. Clodic A. Alami R. (2014). A framework for endowing an interactive robot with reasoning capabilities about perspective-taking and belief management, in Robot and Human Interactive Communication, 2014 RO-MAN: The 23rd IEEE International Symposium on (Edinburgh: IEEE), 1103–1109.

[B50] Morin A. (2006). Levels of consciousness and self-awareness: a comparison and integration of various neurocognitive views. Conscious. Cogn. 15, 358–371. 10.1016/j.concog.2005.09.006 16260154

[B51] Mutlu B. Terrell A. Huang C.-M. (2013). Coordination mechanisms in human-robot collaboration, in Proceedings of the Workshop on Collaborative Manipulation, 8th ACM/IEEE International Conference on Human-Robot Interaction (Tokyo), 1–6.

[B52] Newell A. (1990). Unified Theories of Cognition. Cambridge, MA: Harvard University Press.

[B53] Nikolaidis S. Shah J. (2012). Human-robot teaming using shared mental models, in IEEE/ACM International Conference on Human-Robot Interaction, Workshop on Human-Agent-Robot Teamwork (Boston, MA).

[B54] Oizumi M. Albantakis L. Tononi G. (2014). From the phenomenology to the mechanisms of consciousness: integrated information theory 3.0. PLoS Comput. Biol. 10:e1003588. 10.1371/journal.pcbi.1003588 24811198 PMC4014402

[B55] Pandey A. K. Alami R. (2014). Towards human-level semantics understanding of human-centered object manipulation tasks for HRI: reasoning about effect, ability, effort and perspective taking. I. J. Soc. Robot. 6, 593–620. 10.1007/s12369-014-0246-y

[B56] Pandey A. K. Ali M. Alami R. (2013). Towards a task-aware proactive sociable robot based on multi-state perspective-taking. I. J. Soc. Robot. 5, 215–236. 10.1007/s12369-013-0181-3

[B57] Papon J. Abramov A. Schoeler M. Worgotter F. (2013). Voxel cloud connectivity segmentation - Supervoxels for point clouds, in Proceedings of the IEEE Computer Society Conference on Computer Vision and Pattern Recognition (Portland, OR), 2027–2034.

[B58] Pezzulo G. Donnarumma F. Dindo H. (2013). Human sensorimotor communication: a theory of signaling in online social interactions. PLoS ONE 8:e79876. 10.1371/journal.pone.0079876 24278201 PMC3835897

[B59] Prinz W. (1997). Perception and action planning. Eur. J. Cogn. Psychol. 9, 129–154. 10.1080/713752551

[B60] Renaudo E. Devin S. Girard B. Chatila R. Alami R. Khamassi M. (2015a). Learning to interact with humans using goal-directed and habitual behaviors, in RoMan 2015, Workshop on Learning for Human-Robot Collaboration (Kobe).

[B61] Renaudo E. Girard B. Chatila R. Khamassi M. (2014). Design of a control architecture for habit learning in robots, in Biomimetic and Biohybrid Systems, LNAI Proceedings (Milan), 249–260. 10.1007/978-3-319-09435-9_22

[B62] Renaudo E. Girard B. Chatila R. Khamassi M. (2015b). Respective advantages and disadvantages of model-based and model-free reinforcement learning in a robotics neuro-inspired cognitive architecture, in Biologically Inspired Cognitive Architectures BICA 2015, (Lyon), 178–184.

[B63] Renaudo E. Girard B. Chatila R. Khamassi M. (2015c). Which criteria for autonomously shifting between goal-directed and habitual behaviors in robots?, in 5th International Conference on Development and Learning and on Epigenetic Robotics (ICDL-EPIROB) (Providence, RI), 254–260.

[B64] Richardson M. J. Marsh K. L. Isenhower R. W. Goodman J. R. Schmidt R. C. (2007). Rocking together: dynamics of intentional and unintentional interpersonal coordination. Hum. Movem. Sci. 26, 867–891. 10.1016/j.humov.2007.07.002 17765345

[B65] Rizzolatti G. Sinigaglia C. (2010). The functional role of the parieto-frontal mirror circuit: interpretations and misinterpretations. Nat. Rev. Neurosci. 11, 264–274. 10.1038/nrn2805 20216547

[B66] Sahin E. Cakmak M. Dogar M. R. Ugur E. Ucoluk G. (2007). To afford or not to afford: a new formalization of affordances toward affordance-based robot control. Adapt. Behav. 15, 447–472. 10.1177/1059712307084689

[B67] Seth A. K. Suzuki K. Critchley H. D. (2012). An interoceptive predictive coding model of conscious presence. Front. Psychol. 2:395. 10.3389/fpsyg.2011.00395 22291673 PMC3254200

[B68] Shadlen M. N. Kiani R. (2011). Consciousness as a decision to engage, in Characterizing Consciousness: From Cognition to the Clinic?, eds Dehaene S. Christen Y. (Berlin; Heidelberg: Springer), 27–46.

[B69] Shoda Y. LeeTiernan S. Mischel W. (2002). Personality as a dynamical system: emergence of stability and distinctiveness from intra and interpersonal interactions. Pers. Soc. Psychol. Rev. 6, 316–325. 10.1207/S15327957PSPR0604_06

[B70] Silberman N. Hoiem D. Kohli P. Fergus R. (2012). Indoor segmentation and support inference from RGBD images, in Proceedings of the 12th European Conference on Computer Vision (Florence), 746–760.

[B71] Sisbot E. A. Alami R. (2012). A human-aware manipulation planner. IEEE Trans. Robot. 28, 1045–1057. 10.1109/TRO.2012.2196303

[B72] Sisbot E. A. Marin-Urias L. F. Alami R. Siméon T. (2007). A human aware mobile robot motion planner. IEEE Trans. Robot. 23, 874–883. 10.1109/TRO.2007.904911

[B73] Sisbot E. A. Ros R. Alami R. (2011). Situation assessment for human-robot interactive object manipulation, in RO-MAN, 2011 IEEE (Atlanta, GA: IEEE), 15–20.

[B74] Staudte M. Crocker M. W. (2011). Investigating joint attention mechanisms through spoken human–robot interaction. Cognition 120, 268–291. 10.1016/j.cognition.2011.05.005 21665198

[B75] Sutton R. S. Barto A. G. (1998). Reinforcement Learning: An Introduction, Vol. 1 Cambridge: MIT Press.

[B76] Tomasello M. Carpenter M. (2007). Shared intentionality. Dev. Sci. 10, 121–125. 10.1111/j.1467-7687.2007.00573.x 17181709

[B77] Tomasello M. Carpenter M. Call J. Behne T. Moll H. (2005). In search of the uniquely human. Behav. Brain Sci. 28, 721–727. 10.1017/S0140525X05540123 16262930

[B78] Van Gulick R. (2017). Consciousness, in The Stanford Encyclopedia of Philosophy, Summer 2017 Edn., ed Zalta E. N. (Metaphysics Research Lab, Stanford University). Available online at: https://plato.stanford.edu/entries/consciousness/

[B79] van Hoof H. Kroemer O. Peters J. (2014). Probabilistic segmentation and targeted exploration of objects in cluttered environments. IEEE Trans. Robot. 30, 1198–1209. 10.1109/TRO.2014.2334912

[B80] van Ulzen N. R. Lamoth C. J. Daffertshofer A. Semin G. R. Beek P. J. (2008). Characteristics of instructed and uninstructed interpersonal coordination while walking side-by-side. Neurosci. Lett. 432, 88–93. 10.1016/j.neulet.2007.11.070 18242846

[B81] Vesper C. (2014). How to support action prediction: evidence from human coordination tasks, in Robot and Human Interactive Communication, 2014 RO-MAN: The 23rd IEEE International Symposium on (Edinburgh: IEEE), 655–659.

[B82] Viejo G. Khamassi M. Brovelli A. Girard B. (2015). Modeling choice and reaction time during arbitrary visuomotor learning through the coordination of adaptive working memory and reinforcement learning. Front. Behav. Neurosci. 9:225. 10.3389/fnbeh.2015.00225 26379518 PMC4549628

[B83] Wimmer H. Perner J. (1983). Beliefs about beliefs: representation and constraining function of wrong beliefs in young children's understanding of deception. Cognition 13, 103–128. 10.1016/0010-0277(83)90004-5 6681741

[B84] Yin H. H. Knowlton B. J. (2006). The role of the basal ganglia in habit formation. Nat. Rev. Neurosci. 7, 464–476. 10.1038/nrn1919 16715055

